# Antifungal Natural Products Originating from Endophytic and Rhizospheric Microbes Isolated from Coastal Vegetation

**DOI:** 10.3390/jox15010032

**Published:** 2025-02-17

**Authors:** Sumali Lakmini Dissanayake Jayaweera, Thi Thu Hao Van, Daniel Anthony Dias

**Affiliations:** 1 School of Science, RMIT University, Bundoora, VIC 3083, Australia; s3762179@student.rmit.edu.au (S.L.D.J.); thithuhao.van@rmit.edu.au (T.T.H.V.); 2 ARC Training Centre for Hyphenated Analytical Separation Technologies (HyTECH), CASS Food Research Centre, School of Exercise and Nutrition Sciences, Deakin University, Burwood, VIC 3125, Australia

**Keywords:** antifungals, endophytes, rhizospheric microbes, coastal plants, mangroves, salt marshes, seagrasses, sand dune plants

## Abstract

*Candida* infections severely impact patients who are immunocompromised. Currently, there are limited options to treat fungal infections, especially drug-resistant-fungal infections. Therefore, investigating alternative or repurposed antifungals is paramount. Endophytic microbes (EMs) and rhizospheric microbes (RMs) emerge as promising reservoirs of bioactive natural compounds. Interestingly, plants that have adapted to various environmental conditions harbour a plethora of microbes producing a variety of bioactive natural products that can be assessed for potential antifungal activity. To date, EMs and RMs residing in coastal plants and their associated antifungals have not been extensively studied or reviewed. Therefore, this comprehensive review will focus on antifungal natural products, extracted from coastal-vegetation-associated microbiota to draw the attention of research in this field. A comprehensive literature search was conducted by examining both Scopus and Google Scholar databases during the period of 2013–2024 related to the following coastal vegetation: mangroves, sand dune plants, salt marsh plants, and seagrasses. To date, 65 novel antifungal compounds derived from coastal-plant EMs and RMs have been identified. Mangroves were found to be the most prominent host harbouring antifungal-producing EMs and RMs compared with other coastal plants. Coastal-plant-associated fungal partners were the most prominent producers of antifungals compared to their bacterial counterparts. Fifty-four fungal-EM/RM derived antifungals have been reported to demonstrate activities against plant pathogenic fungi as well as human fungal pathogens. Most of the bacterial-derived antifungals (11 antifungals) have previously been reported to have antifungal activity against *Candida albicans*.

## 1. Introduction

Fungal diseases refer to a type of infectious disease caused by unicellular or multicellular eukaryotic organisms known as fungi [1,2,3]. The primary fungal taxa associated with infecting healthy human hosts include Ascomycota, Basidiomycota, and the subdivision Entomophthoromycotina [4]. Among these, Ascomycota includes several common and severe disease-causing fungi such as *Candida* sp., *Aspergillus* sp., and *Histoplasma* sp., and dermatophytosis-causing fungi such as *Trichophyton* sp., *Microsporum* sp., and *Epidermophyton* sp. [5,6]. The impact of fungal infections ranges from localised, external, mild skin, nail, and hair infections to more severe infections impacting internal organs such as the lungs, gastrointestinal tract, blood, and brain. More than 300 million people are affected by serious fungal diseases worldwide, which lead to 1.6 million deaths annually [7,8]. Among them, approximately 90% of deaths are due to Candidiasis, Aspergillosis, and Cryptococcosis [9]. The severity of fungal diseases and the clinical outcome depend on risk factors of the host, including a weak or compromised immune system [10], pathogen virulence (e.g., dimorphism, fungal cellular structures such as cell walls and cell membranes) and therapeutic factors such as side effects due to the administering of antifungal drugs [11].

Currently, there are several antifungal drug classes available: azoles (fluconazole, voriconazole, posaconazole, itraconazole, clotrimazole, and ketoconazole), echinocandins (caspofungin, anidulafungin, and micafungin), polyene antifungals (amphotericin B, nystatin, and natamycin), pyrimidine analogues (flucytosine), allylamines (naftifine and terbinafine), oxaborole (tavaborole), and thiocarbamate (tolnaftate) [12,13,14]. Primarily, azole-based drugs are the first line of treatment [10]. Azoles are chemically synthesised drugs that hinder the biosynthesis of ergosterol, which is an important sterol in the production of fungal cell membranes. Azoles interrupt ergosterol biosynthesis by inhibiting the enzyme called lanosterol-14α-demethylase, resulting in the accumulation of lanosterol in fungal cells. Lanosterol can convert to 14α-methyl-3,6-dienol, which is toxic to fungi [11,15,16]. The echinocandin family of drugs is important for treating invasive fungal diseases. Echinocandins are cyclic hexapeptides involved in the non-competitive inhibition of a membrane-bound enzyme called *β*-1,3-glucan synthase, which is an important enzyme in the biosynthesis of the major fungal cell wall carbohydrate called *β*-1,3-glucan, which affects the integrity of the cell wall, resulting in cell damage [16,17]. Polyenes are broad-spectrum antifungals used to treat infections caused by most yeasts and filamentous and dimorphic fungi [12]. Polyenes bind to ergosterol, which results in the disruption of the cell membrane, leading to an increase in membrane permeability and ultimately cell death [7].

At present, a major problem related to antifungals is the development of antifungal drug-resistant fungal strains, leading to complications in curing fungal diseases using first-line antifungals. The prolonged use of antifungal therapies may lead to selecting existing intrinsically drug-resistant fungal strains over susceptible strains or induce generations of acquired drug resistance (i.e., by means of mutations or horizontal gene transfer) in some strains [11]. One mutation of azole-based antifungal drug resistance in *Candida* involves mutation in the *ERG 11* gene, which encodes lanosterol the 14α-demethylase enzyme located on the fifth chromosome. These mutations can reduce the enzyme’s affinity for azole-based antifungals or lead to its upregulated production. For example, *Pichia kudriavzeveii* (formerly *Candida krusei*) [18] inherently possesses such a mutation, whereas some strains of *C. albicans*, *Candida parapsilosis,* and *Candida tropicalis* have acquired *ERG 11* mutations, resulting in similar resistance [11]. Another resistance mechanism is the increased expression of efflux pumps, such as the ATP-binding cassette family transporter pump (encoded by the *Candida* drug resistance gene—*CDR*) and major facilitator superfamily pumps (encoded by multidrug resistance genes—*MDR*). These pumps use ATP or the electrochemical proton gradient to actively transport substances across the plasma membrane—for instance, the removal of antifungal agents out of the cell [11,19]. Overexpression of *CDR* genes has been observed in *C. albicans*, *Nakaseomyces glabrata* (formerly *Candida glabrata*) [18], and *P. kudriavzeveii*, while increased expression of *MDR* genes has been reported in *C. albicans* and *C. parapsilosis* [11,20].

Polyene-base antifungal drug resistance is uncommon compared to azole-based antifungal drug resistance. One of the two main mechanisms is the replacement of ergosterol with other ergosterol intermediates such as fecosterol or episterol along with a reduction in ergosterol biosynthesis. The alternate mechanism is to increase ergosterol biosynthesis in order to manage the concentration of polyene-based antifungal drugs. *Clavispora lusitaniae* (formerly *Candida lusitaniae*), *N. glabrata*, and *Meyerozyma guilliermondii* (formerly *Candida guilliermondii*) have been reported to have amphotericin B resistance, while *C. parapsilosis* has resistance against natamycin [18,21,22].

Echinocandin-based antifungal drug resistance of *Candida* is due to point mutations in *FKS 1* or *FKS 2*, which causes conformational changes in the binding sites of *β*-1,3-glucan synthase to echinocandin-based antifungal drugs [11]. A natural *FKS1* mutation has been observed in *C. parapsilosis* and *M. guillermondii*, and an acquired *FKS1* mutation due to prolonged echinoandin-based antifungal drug usage has been detected in *C. albicans*, *C. tropicalis*, *P. kudriavzeveii*, and *N. glabrata,* whereas an *FKS2*-acquired mutation has been reported in *N. glabrata* [11].

The emergence of antifungal resistance poses a serious threat to immunocompromised patients in hospitals [11]. Furthermore, some antifungal drugs can cause various side-effects, including skin irritations, gastrointestinal disturbances, nephrotoxicity, and liver dysfunction [12,23]. Consequently, there is a pressing requirement for new drugs to overcome these constraints. An ideal antifungal drug should be broad-spectrum, target novel mechanisms to inhibit resistant fungal pathogen strains, have minimal side effects, and be available in different delivery methods, such as oral and intravenous routes [7,19]. According to Newman and Cragg, during the period of 2011 to 2019, four antifungals (i.e., efinaconazole, tavaborole, isavuconazonium sulfate, and fosravuconazole) were approved, all of which were synthetically produced [24,25].

However, there are some novel antifungals in the clinical environment with new or modified molecular structures that show promise in addressing issues related to current antifungal drugs. Rezafungin (formerly known as CD101) is a semi-synthetic cyclic hexapeptide derived from a fermented product of *Aspergillus nidulans*. It is an analogue of capsofungin and anidulafungin, which are natural products of the fungi *Glarea lozoyensis* and *Aspergillus nidulans*, respectively. Rezafungin exhibits improved characteristics such as a prolonged half-life and the absence of producing toxic intermediates [26,27,28]. An alternate, promising antifungal is VL-2397 (formerly ASP2397), a cyclic hexapeptide that is a hydroxamate siderophore isolated from *Acrmonium persicinum*. VL-2397 displays antifungal properties against *Aspergillus* and some *Candida* sp. [29,30]. It is capable of being transported into fungal cells through the cell membrane via the siderophore iron transporter 1, which is not present in mammalian cells. However, the precise mechanism of its antifungal activity is still not fully understood [28]. Ibrexafungerp (formerly MK-3118 and SCY-078) is a semi-synthetic triterpenoid that targets the *β*-1,3-glucan synthase activity. It can be administrated orally and is effective against biofilm-producing *Candida* sp. and antifungal-resistant strains displaying FKS1 and FKS2 mechanisms [11,28]. BSG005 is a polyene antifungal similar to nystatin A, isolated from *Streptomyces noursei*. It possesses antifungal activity against azole- and echinocandin-resistant *Aspergillus* and *Candida* sp., with lower nephrotoxicity compared to other polyenes [28]. These potential drugs, which are either semi-synthetic or of natural product origin, highlight the importance of exploring new chemical scaffolds with potential antifungal bioactivities, an approach that can lead to the discovery and development of new antifungal drugs [11,31]. Natural products, especially those originating from plant-beneficial microbial communities such as EMs and RMs, are a promising source of bioactive natural products. Endophytes live within the host’s intercellular spaces, such as vascular bundles of plants, without causing any immediate negative impact to the host. RMs are plant-root-associated microbes [32] that reside within the root zone of soil. Fungi and bacteria are commonly occurring EMs and RMs in plants. Among these, fungal endophytes are more frequently isolated and often engage in mutualistic relationships [32,33]. Nevertheless, under favourable conditions, they may occasionally adopt parasitic or pathogenic behaviours. These instances arise when host immunity is minimal or when unfavourable environmental conditions impede host growth [33]. Fungal EMs and RMs typically fall within the taxonomic divisions of Ascomycota, occasionally Zygomycetes, and Basidiomycetes [32]. Bacterial EMs and RMs can be found within the phylum Actinomycetota (formerly Actinobacteria), exemplified by species like *Streptomyces* sp.; within the phylum Pseudomonadota (formerly Proteobacteria), represented by species such as *Pseudomonas* sp.; and within the phylum Bacillota (formerly Firmicutes), typified by organisms like *Bacillus* sp. [32,34,35].

**Figure 1:**
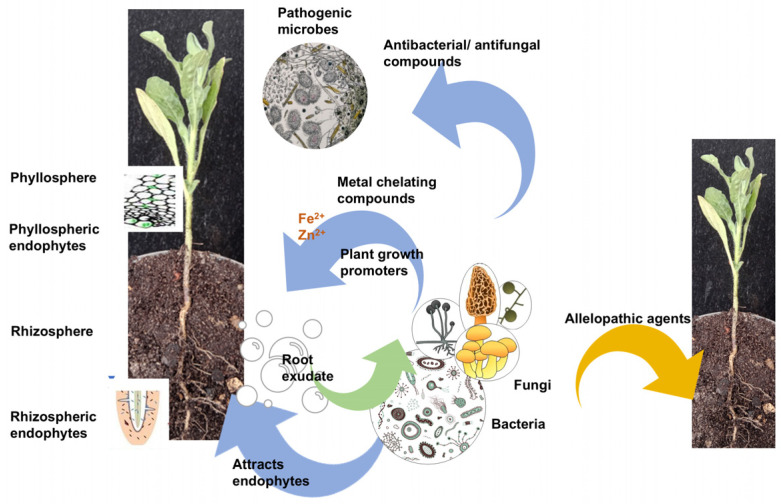
Some interactions that strengthen plant–microbial association. Adapted from [45].

When these microorganisms engage in mutualistic relationships, they obtain their nutrition and protection from the host organism (Figure 1). Conversely, certain endophytes can enhance the inorganic nutrient uptake for the host. For instance, the endophyte *Candidatus Celerinatantimonas neptuna* in *Posidonia oceanica* aids in nitrogen fixation [36]. Additionally, some endophytes biosynthesise vital hormones, such as indole acetic acid produced by *Ralstonia* and *Paenibacillus* sp., which are seed endophytes of the salt marsh plant *Cistanche phelypaea* [37].Characteristic high-salinity conditions in a coastal ecosystem is a significant abiotic factor that limits plant growth performance due to osmotic stress and oxidative damage in coastal plants [38]. This stress also increases susceptibility to bacterial and fungal pathogens, as observed in studies on salt-sensitive plants [39]. However, inoculating salt-sensitive plants with halotolerant, antifungal-producing endophytic microbes under saline conditions has shown positive effects such as enhanced growth, management of salt stress, and improved biological control capabilities [38,40,41,42]. For instance, the salt marsh plant *Phragmites australis*, along with the rhizospheric fungus *Trichoderma arenarium*, coexist to provide defence against plant pathogens such as *Rhizotonia solani* and *Alternaria alternata* [42]. Additionally, some endophytes play a key role in mitigating stress conditions by producing numerous types of natural products. For example, the *Glutamicibacter halophytocola* bacterium strain KLBMP 5180, isolated from the roots of the coastal plant *Limonium sinense*, was re-inoculated into *L. sinense* seedlings grown under sterile, saline conditions with 250 mM NaCl. This inoculation resulted in significant seedling growth and upregulation of flavonoid and phenylpropanoid biosynthetic pathways in the leaves of inoculated plants compared to non-inoculated plants [43]. Thus, EMs and RMs found in coastal vegetation are capable of tolerating coastal environmental stress conditions [44]. These microorganisms play a crucial role in supporting the thriving of coastal plants within these ecosystems through their symbiotic interactions [43].

These residing microbes have the capability to biosynthesise their own NPs that are bioactive [42,46]. These bioactive compounds produced by plant-beneficial microbial-communities provide additional defence mechanisms against plant pathogens, particularly for plants thriving in saline environments. These associations warrant further investigation to identify important NPs for potential antifungal activity screening. The antifungal properties of microbial communities associated with mangroves have been studied better in comparison to the other coastal plants [47]. This comprehensive review aims to examine recent research on novel antifungal NPs derived from microbiota associated with coastal vegetation, while encouraging researchers to investigate antifungal NPs from diverse coastal vegetation. Importantly, this review examines the molecular structural moieties responsible for antifungal activity by comparing structurally closely related compounds that exhibit varying degrees of antifungal efficacy, including those that are highly antifungal, moderately antifungal, and non-antifungal.

## 2. Materials and Methods

A comprehensive literature search was conducted utilising the following keywords to gather relevant information. The keywords included natural products, coastal vegetation/plants, mangroves, salt marshes, sand dunes, seagrasses, endophytes, and rhizospheric microorganisms such as fungi, bacteria, and actinomycetes, as well as antifungal natural products. This search was conducted across the Scopus database and Google Scholar. Scientific publications published within the last 10 years were examined, with a focus on studies involving coastal-plant-associated EMs and RMs that produce novel antifungal NPs. The studies that involved in vitro bioassays for assessing the antifungal properties of these natural products were selected. Moreover, studies exploring antifungal compounds against fungal phytopathogens were also included. The studies with documented antifungal compounds that have already been reported were excluded.

During the preparation of this manuscript, the authors used Photoshop 2025, AI Generative Fill tool for the purposes of creating the background and few objects within the graphical abstract.

## 3. Results and Discussion

### 3.1. Tropical Coastal Vegetation and Its Environmental Adaptations

Coastal ecosystems are one of the largest ecosystems in the world and serve as a crucial transitional zone between oceanic and terrestrial ecosystems. These ecosystems encompass diverse halophytic plants that have adapted to extreme conditions. Coastal plant communities exhibit remarkable resilience to challenges such as soil salinity, low water availability, salt spray, high wind velocity, sandstorms, wave action, water table fluctuations, and periods of flooding. Mangroves, salt marsh plants, sand dune plants, and seagrasses [48] are some examples of common coastal vegetation.

Mangrove plants, although terrestrial by nature, have evolved to thrive specifically in coastal ecosystems. Their distribution spans approximately 137,600 km^2^ across 118 countries worldwide [49]. Mangroves thrive in their environment, possessing morphological and functional adaptations enabling them to overcome coastal environmental constraints [50]. For example, mangroves have leaves with salt-secreting glands (e.g., *Aegiceras corniculatum*) and germinated seeds, known as droppers, that can submerge themselves in mud and propagate or disseminate through water. Furthermore, specialised root systems include prop roots (e.g., *Rhizophora apiculata*), buttress roots (e.g., *Xylocarpus granatum*), knee roots (e.g., *Bruguiera gymnorrhiza* and *B. cylindrica*), and cone roots (e.g., *Avicennia* sp.) that improve physical stability in muddy soil and facilitate root ventilation [51].

On the other hand, salt marshes are coastal wetlands characterised by occasional and seasonal tidal activities, exhibiting resilience to saline, waterlogging, and low-oxygen environmental conditions. Salt marshes appear to be intermittently distributed, and are substituted by mangroves in tropical and sub-tropical regions. Plant species belonging to families such as Amaranthaceae (*Arthrocnemum indicum*), Poaceae (*Porteresia coarctata*), Cyperaceae (*Fimbristylis ferruginea*), Boraginaceae (*Heliotropium curassavicum*), and Convolvulaceae (*Cressa cretica*) [52] can be found in tropical salt marshes.

Sand dunes are formed by loose sand deposited around coastal zones. Sand dune plant species composition and diversity may vary depending on their distance from the shoreline [53,54]. These plants exhibit resilience to arid and nutrient-stressed coastal habitat conditions including salt spray, high wind velocity, and high temperatures [54]. *Ipomoea pescaprae*, *Ipomoea imperati*, *Casuarina equisetifolia*, *Terminalia catappa*, *Hibiscus tiliaceus*, *Premna serratifolia*, *Anthemis maritima* L., and *Elymus farctus* are some examples of plant species inhabiting sand dunes [53,54].

Seagrasses, categorised as angiosperms or flowering plants, growing in submerged habitats within shallow coastal waters, span approximately 136 countries in tropical and subtropical regions [55]. These plants display adaptability to saline water conditions, thriving under submerged environments with highly functional roots in anaerobic soils. Seagrasses utilise gas-filled intercellular spaces known as lacunae to transport oxygen from leaves to roots and rhizomes, ensuring their oxygen requirements are met, facilitating leaf buoyancy for optimal light exposure by minimising mutual shading. The leaf epidermis lacks stomata and is rich in chlorophyll, unlike the mesophyll. Additionally, the epidermis is covered with a thin cuticle layer to enhance permeability to water, gases, and solute [56]. Globally, seagrass meadows encompass 66 species belonging to plant families such as Cymodoceaceae (*Amphibolis griffithii*, *Cymodocea angustata*), Posidoniaceae (*Posidonia australis*), Hydrocharitaceae (*Halophila australis*), and Zosteraceae (*Zostera mulleri*) [56,57].

### 3.2. Endophytic/Rhizospheric Microbial Diversity in Coastal Vegetation

#### 3.2.1. Fungal Diversity

Among coastal plant-associated fungal species, the majority of them belong to the division of Ascomycota (Table 1) [58,59]. Among these, *Penicillium* sp. (order Eurotiales) and *Fusarium* sp. stand out as the most frequently isolated fungal species linked with coastal plants. Additionally, *Penicillium*, *Aspergillus* (order Eurotiales), and *Fusarium* sp. (order Hypocreales) exhibit notable diversity in species compared to other reported genera [59,60]. Remarkably, mangrove species such as *Acanthus ilicifolius*, *Aegiceras corniculatum*, *Acrostichum aureum*, and *Avicennia marina* emerge as a significant reservoir of fungal EMs and RMs harbouring gr eater diversity [61].

#### 3.2.2. Bacterial Diversity

Previous investigation into endophytic and rhizospheric bacteria has received comparatively less attention than studies on fungi. However, as presented in Table 2, there have been more examples of endophytic bacteria associated with coastal plants. Prominent bacterial phyla identified in coastal plants include Pseudomonadota, Bacillota, Actinomycetota, and Bacteroidota (previously known as Bacteroidetes) (Table 2) [35,62,63]. Notable bacterial genera observed encompass *Erwinia*, *Vibrio*, *Psychrobacter*, *Paenibacillus*, *Enterococcus*, *Streptomyces*, *Aidingimonas*, *Marinobacter*, *Chromohalobacter*, *Bacillus*, and *Isoptericola*, and *Bacillus* sp. such as *B. safensis*, *B. licheniformis*, and *B. subtilis* [63,64]. Among these bacteria, the most promising natural products producing bacterial groups belong to the phylum Actinomycetota. Actinomycetes, members of the class Actinomycetia [65,66], have adapted to a wide range of terrestrial and aquatic environments even under extreme environmental conditions [67]. *Streptomyces*, belonging to the order Streptomycetales and the family *Streptomycetaceae*, is the predominant culturable endophytic genus among actinomycetes, and it is the most prolific producer of secondary metabolites, according to the pioneering research by Selman Waksman in 1943 [35,68,69]. However, *Nocardia*, *Streptosporangium*, *Nocardioides*, *Pseudonocardia*, *Microbacterium*, *Actinomadura*, *Actinocorallia*, *Saccharopolyspora*, *Curtobacterium*, *Ilumatobacter*, *Herbiconiux*, *Dietzia*, *Kineococcus*, *Citriococcus*, *Janibacter*, and *Micromonospora* are other endophytic genera belonging to the phylum Actinomycetota that are also found among coastal plants. These genera are considered rare actinomycetes due to their infrequent isolation and challenges associated with laboratory cultivation [70,71,72,73]. Experiments conducted by Chen, Zhang [73] reported a three-fold higher diversity of Actinomycetota in culture-independent bacterial studies carried out using 16S rRNA gene-based high-throughput sequencing methods compared to culture-dependent bacterial studies utilising actinomycetes-selective culture media.

### 3.3. Production of Antifungals from Associated Coastal Plant Microorganisms

Coastal plants have adapted to thrive in extreme and unique environmental conditions and alternatively, most of them hold ethnomedicinal significance, as they are rich sources of natural products [74]. Secondary metabolites such as alkaloids, flavonoids, terpenoids, triterpenoid saponins, and quinones exhibit antifungal properties [74,75,76]. Traditionally, in various countries, *X. granatum* (cannonball mangrove, cedar mangrove) has been used to treat diarrhoea and thrush (*Candida* infection) [75]. The fungal endophyte *Phomopsis* sp. MA125 (GU592014) isolated from *X. granatum* displayed antifungal activity against *Candida albicans* ATCC90028 [77]. The stems of the sand dune plant *Salvadora persica* (family salvadoraeceae) have been used as miswak (toothbrush) in traditional Middle Eastern countries for oral hygiene [78]. Endophytic species derived from *S. persica*, namely, *Penicillium restrictum*, *Penicillium citrinum*, and *Penicillium canescens* and fluorescent *Pseudomonas* species, exhibited antifungal activity against phytopathogens such as *Macrophomina phaseolina*, *Rhizoctonia solani*, *Fusarium solani*, and *Fusarium oxysporum* [79,80]. *Posidonia oceanica* (L.) is a seagrass species belonging to the family Posidoniaceae. In Tunisia, *Posidonia oceanica* leaves have been utilised as livestock bedding due to their antifungal and insect repellent properties [81]. In another study, the Ascomycota *Mariannaea humicola* IG100, isolated from *Posidonia oceanica* collected from the Tyrrhenian Sea, displayed antifungal activity against *Aspergillus flavus* IG133, *Penicillium griseofulvum* TSF04, and *Trichoderma pleuroticola* IG137 due to the terpenoid terpestacin 1 [82].

**Figure 2:**
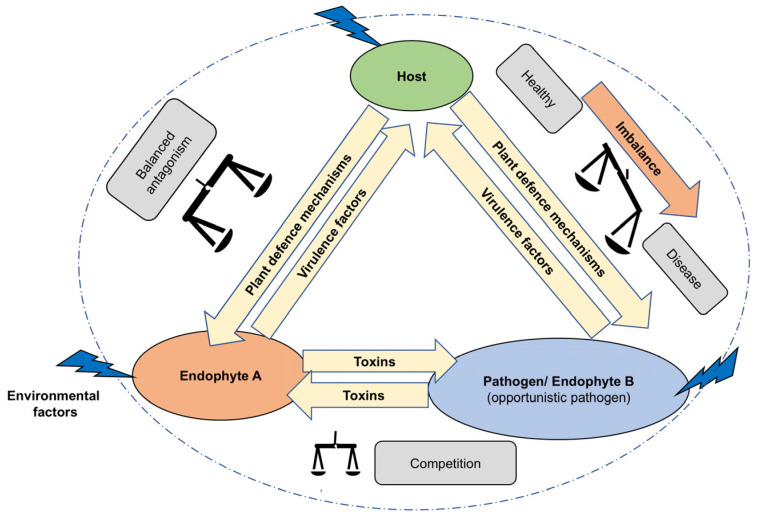
Balanced antagonism maintained by microbial communities of the host plant, their coexistence, and plant survival. Adapted from [84].

A single plant tissue may harbour a diverse range of microbes or microbial communities, some of which act as beneficial mutualistic partners, while some of the others can be harmful pathogens. These microorganisms engage in communication with neighbouring microbes (i.e., a bacteria, fungus, or virus) and the host plant, known as microbial interactions. Several possible interactions include the interaction between endophyte A and the host, the interaction between endophyte A and endophyte B or a pathogen, and the interaction involving endophyte B or a pathogen and the host. These interactions can span a spectrum from mutualism to parasitism or invasion, facilitated by signalling molecules and secondary metabolites. Some specific types of interaction involve the production of antimicrobial compounds (such as antibacterial and antifungal compounds) [83]. Plant endophytic microorganisms produce antifungals to prevent and/or suppress other endophytic fungal competitors from invading their space as well as to protect the host from fungal pathogens [32,33]. The antagonistic role is balanced among the members of the plant microbial community and the host plant (Figure 2) [84]. Apart from this, plant growth and development, and disease management, are weakened due to adverse environmental conditions such as salinity in coastal ecosystems [38,39]. In coastal ecosystems, the presence of these mutualistic microbial communities and their antagonistic agents offers an extra line of defence, strengthening disease management [38]. The subsequent sections will discuss novel antifungal natural products derived from endophytes associated with coastal vegetation.

### 3.4. Antifungals from Endophytic/Rhizospheric Fungi

#### 3.4.1. Naphthalene Derivatives

**Figure 3:**
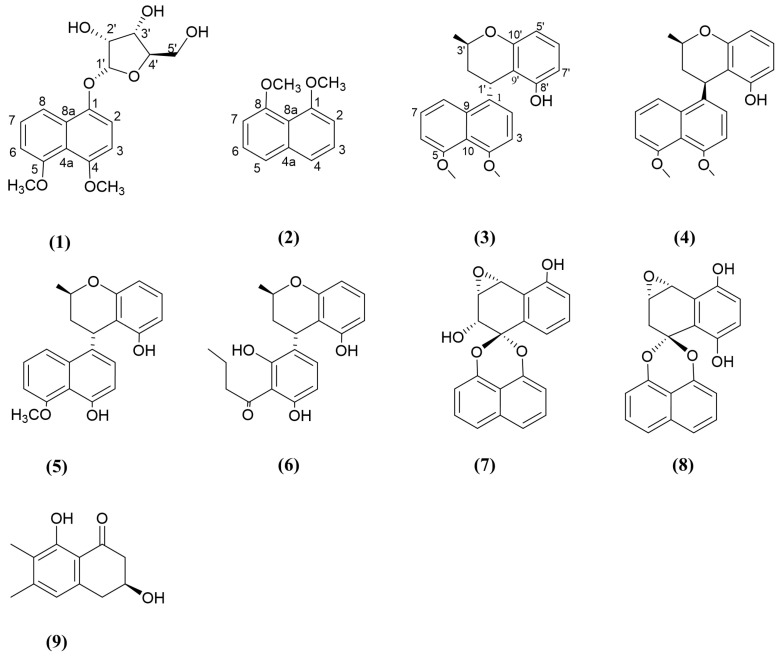
Compounds 1–9: (1) nalesconosides A, (2) 1,8-dimethoxynaphthalene, (3) cladonaphchroms A, (4) cladonaphchroms B, (5) nodulisporins D, (6) nodulisporins F, (7) guignardin B, (8) cladospirone F, (9) (3S)-3,8-dihydroxy-6,7-dimethyl-α-tetralone.

Natural naphthalene derivatives are polyketides consisting of two benzene rings that are fused together at the ortho position [85]. Naftifine, tolnaftate, and terbinafine are naphthalene-derived antifungal drugs available on the market [86]. An endophytic fungus, *Daldinia eschscholzii* MCZ-18, isolated from a healthy branch of *Ceriops tagal,* was used to extract five novel naphthalene derivatives. Among them, dalesconosides A (Table S1, Figure 3, compound 1) featured a rare ribofuranoside substitution at C-1 and displayed an antifungal effect against *C. albicans*, with an MIC value of 25 μg/mL. In comparison, the positive control, amphotericin B, displayed an MIC value of 0.78 μg/mL [87]. The ribofuranoside connected to C-1 via an oxygen atom is hypothesised to contribute to the antimicrobial activity of dalesconosides A, as opposed to 1,8-dimethoxynaphthalene (Figure 3, compound 2), a known compound from the same experiment, which displayed an MIC value of 50 μg/mL against *C. albicans*. In a study by Ma, Zheng [88], two new isomers of naphthalene chroman-coupling derivatives, called cladonaphchroms A (Figure 3, compound 3) and B (Figure 3, compound 4), were isolated from a *Ceriops tagal* (mangrove species)-derived fungus called Cladosporium sp. JJM22. These two compounds have shown antifungal activities against *Alternaria brassicicola*, *Phytophthora parasitica* var. nicotianae, *Colletotrichum capsici*, *Bipolaris oryzae*, *Diaporthe medusaea* Nitschke, and *Ceratocystis paradoxa* Moreau (Table S1) [88]. The MIC values (50 μg/mL) of both cladonaphchroms A and B against *Phytophthora parasitica* were similar to those of the positive control, Prochloraz. Furthermore, the potency of the antifungal activity of cladonaphchroms A against *Diaporthe medusaea* was comparable to that of the positive control (MIC value 50 μg/mL), while cladonaphchroms B showed lower antifungal activity (MIC value 100 μg/mL). According to the authors, these compounds share closely related 2D planar structures and molecular weights, displaying a minute difference in the chemical shifts at C-1′/2′/3′9′. These compounds have shown significant differences in antimicrobial activities against different bacterial and fungal pathogens [88]. Further to this, Dai, Krohn [89] published similar molecular structures to compounds 3 and 4, namely, nodulisporins D and F (Figure 3, compounds 5 and 6), which also exhibited antifungal activity. However, the tested pathogens against compounds 5 and 6 and the antifungal susceptibility testing procedures differed from those in Ma, Zheng [88], making it challenging to directly compare the bioactivity of compounds from both studies. Even a small difference in the 3D arrangements of bicyclic naphthalene and chroman groups have shown varying levels of broad-spectrum antifungal and antibacterial activities [88,89]. In a study by Ai, Wei [90], the researchers isolated the endophytic fungus *Guignardia* sp. KcF8 from fresh, healthy fruits of the mangrove *Kandelia candel*, leading to the isolation of six novel compounds. Among them, guignardin B (Figure 3, compound 7), a spirodioxynaphthalene that consists of two 1,8-dihydroxynaphthalene-derived spiroketal units connected through a spiroketal linkage, displayed mild antifungal activities against *Fusarium* sp. and *Aspergillus niger* compared to the positive control, carbendazim [90]. However, the closely related compound cladospirone F (Figure 3, compound 8) with varying positions of having hydroxyl groups, showed no antifungal activity [91]. In a different study, a novel hydronaphthalenone derivative, (*3S*)-3,8-dihydroxy-6,7-dimethyl-*α*-tetralone (Figure 3, compound 9), was identified from the endophytic fungus *Daldinia eschscholtzii* PSUSTD57, isolated from the leaves of the mangrove plant *Bruguiera gymnorrhiza* (L.) in Thailand. This compound exhibited very mild antimicrobial activities against *Microsporum gypseum* (a soil-associated dermatophyte occasionally causing dermatophytosis in humans) [92].

#### 3.4.2. Chromone Derivatives

Chromone is an oxygen-containing heterocyclic compound that has a benzo-γ-pyrone skeleton in which chroman derivatives have been reported to demonstrate a variety of bioactivities [93].

**Figure 4:**
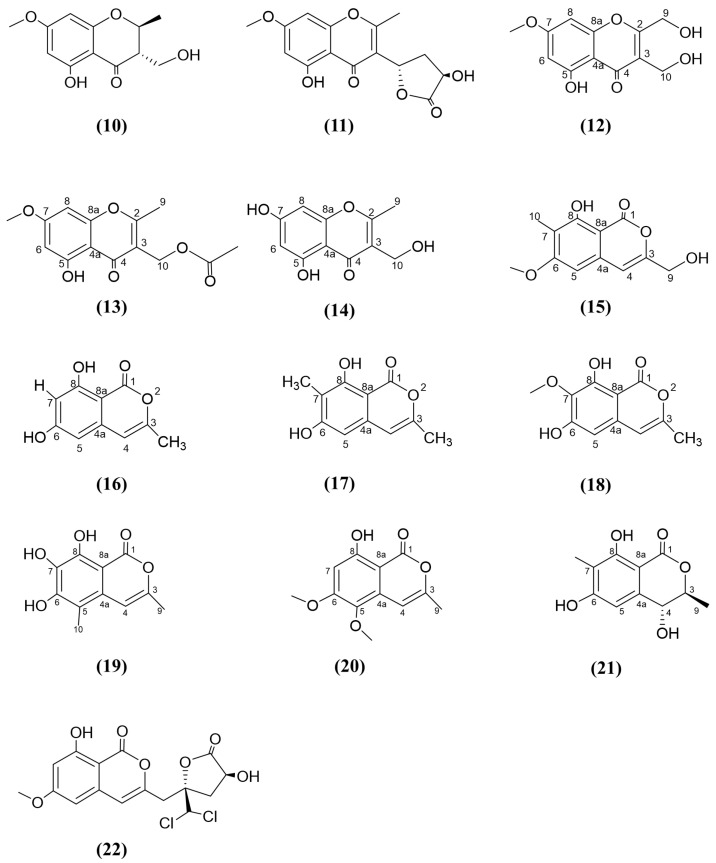
Compounds 10–22: (10) 5-hydroxy-3-hydroxymethyl-7-methoxy-2-methyl-4-chromanone, (11) 5-hydroxy-3-((3′R,5′S)-3′-hydroxy-2′-oxotetrahydrofuran-5′-yl)-7-methoxy-2-methyl-4H-chromen-4-one, (12) 5-hydroxy-2, 3-dihydroxymethyl-7-methoxychromone, (13) 5-hydroxy-3-acetoxymethyl-2-methyl-7-methoxychromone, (14) 5,7-dihydroxy-3-hydroxymethyl-2-methylchromone, (15) 8-hydroxy-3-hydroxymethyl-6-methoxy-7-methylisocoumarin, (16) 6,8-dihydroxy-3-methylisocoumarin, (17) similanpyrone B, (18) reticulol, (19) botryospyrone A, (20) botryospyrone B, (21) botryospyrone C, (22) dichlorodiaportinolide.

The necrotic leaves and healthy roots of the seagrass species *Enhalus acoroides* were used to isolate *Aspergillus alabamensis* SYSU-6778 [94], while the healthy roots were also used to isolate another endophytic fungus, *Aspergillus fumigatiaffinis* SYSU-6786 [95]. A co-culture of these two endophytic fungi led to the isolation of a novel antifungal chromone, 5-hydroxy-3-hydroxymethyl-7-methoxy-2-methyl-4-chromanone (Figure 4, compound 10), which inhibited the growth of *A. alabamensis* with activity comparable to the positive control (MIC value 100 μg/mL), triadimefon [95]. Based on Yin, Tan [96], an undescribed 5-hydroxy-3-(3′R, 5′S)-3′-hydroxy-2′-oxotetrahydrofuran-5′-yl)-7-methoxy-2-methyl-4H-chromen-4-one (Figure 4, compound 11) was extracted from the endophytic fungus *Trichoderma lentiforme* ML-P8-2, isolated from the fresh leaves of *Bruguiera gymnorrhiza.* This compound showed antifungal activity against *C. albicans*, with an MIC value of 15.25 μg/mL, while the positive control, ketoconazole, displayed an MIC value of 0.07 μg/mL [96]. Compounds 10 and 11 are structurally similar, but their antifungal activity cannot be directly compared, as the test strains were different.

Hu and the research team [97] isolated a fungal endophyte, *Botryosphaeria ramosa* L29, from the leaves of *Myoporum bontioides*, a semi-mangrove (i.e., woody plants that grow both on land and in intertidal zones) in Leizhou Peninsula, China. Three new chromones were identified, namely, 5-hydroxy-2,3-dihydroxymethyl-7-methoxychromone (Figure 4, compound 12), 5-hydroxy-3-acetoxymethyl-2-methyl-7-methoxychromone (Figure 4, compound 13), and 5,7-dihydroxy-3-hydroxymethyl-2-methylchromone (Figure 4, compound 14), which displayed antifungal activities against plant pathogenic fungi *F. oxysporum*, *Colletotrichum musae*, *Penicillium italicum*, and *Fusarium graminearum.* Notably, the third chromone, compound 14, demonstrated broad-spectrum antifungal activity, showing potency 16-, three- and six-fold higher than the activity of the positive control, the agricultural fungicide triadimefon, with MIC values of 6.25, 12.5, and 12.5 μg/mL against *F. oxysporum, Penicillium italicum*, and *Colletotrichum musae*, respectively, compared to triadimefon’s MIC values of 100, 50, and 80 μg/mL. Furthermore, compound 12 exhibited seven times (MIC 6.25 μg/mL) the antifungal inhibition against *Penicillium italicum* compared to the same positive control, triadimefon. The enhanced broad-spectrum antifungal activity of compound 14 was due to the presence of the 7-hydroxyl group at C-7, whereas the other two chromones, compounds 12 and 13, contained a 7-methoxy group in place of the 7-hydroxyl group but showed comparatively low antifungal activity to compound 14 [97].

#### 3.4.3. Isocoumarins

Isocoumarins possess the common 1H-2-benzopyran-1-one structure and naturally are derivatives of the polyketide biosynthetic pathway [98,99]. Hu, Wu [97] reported a new isocoumarin, 8-hydroxy-3-hydroxymethyl-6-methoxy-7-methylisocoumarin (Figure 4, compound 15), which displayed antifungal activity against all the aforementioned plant pathogenic fungi tested with the previous chromones (compounds 12, 13, and 14). For compound 15, the presence of a methoxy group in position 6 and/or a 3-hydroxymethyl group in position 3 could be responsible for the antifungal activity compared with other similar isocoumarins, such as 6,8-dihydroxy-3-methylisocoumarin (Figure 4, compound 16), similanpyrone B (Figure 4, compound 17), and reticulol (Figure 4, compound 18), which did not display antifungal activity for *Candida albicans* [97,100]. However, compounds 16, 17, and 18 should be tested against the same fungal pathogens to obtain a more accurate comparison of their respective antifungal activity.

Several new antifungals, including botryospyrone A (Figure 4, compound 19), B (Figure 4, compound 20), and C (Figure 4, compound 21), and (*3aS*,*8aS*)-1-acetyl-1,2,3,3a,8,8a-hexahydropyrrolo[2,3b]indol-3a-ol (discussed in the section on alkaloids), were isolated from the endophytic fungus *Botryosphaeria ramosa* L29, which was isolated from the leaves of *M. bontioides* [101]. Botryospyrone B (compound 20) displayed three and two times higher antifungal activity against *F. oxysporum* and *F. graminearum*, respectively, compared to the positive control, triadimefon. Compound 20 possesses methoxy moieties in positions 5 and 6 and a methyl group at position 3, while compound 15, which was previously discussed, has one methoxy group at position 6 and a 3-hydroxymethyl group in position 3 [97]. Both compounds exhibited antagonism against the same fungal species, but compound 20 showed higher activity compared to compound 15, emphasising the presence of the methoxy group at position 5 and/or the methyl group at position 3 at compound 20 compared to compound 15. The antifungal activity of botryospyrone C (compound 21) was shown to decrease compared to compound 20, likely due to the substitution of the methoxy group at position 6 with a hydroxyl group and the absence of a methoxy group at position 5 in compound 21. Botryospyrone A selectively inhibited *F. oxysporum* with antifungal activity comparable to compound 20. However, compound 20 exhibited strong broad-spectrum antifungal activity. Furthermore, *M. bontioides* A. collected from Leizhou Peninsula, Guangdong province, China, was used to isolate the endophytic *Trichoderma* sp. 09, which subsequently produced a novel chlorine-containing isocoumarin attached to a lactone group (cyclic carboxylic esters), named dichlorodiaportinolide (Figure 4, compound 22). This compound possesses antifungal bioactivity against *Colletotrichum musae* and *Rhizoctonia solani*. Compound 22 displayed antifungal activity against *R. solani* (MIC value 6.25 μg/mL), which was similar to the positive control, carbendazim (Table S1), which is a widely used, systemic, broad-spectrum agricultural fungicide [102].

#### 3.4.4. Ether Compounds

**Figure 5:**
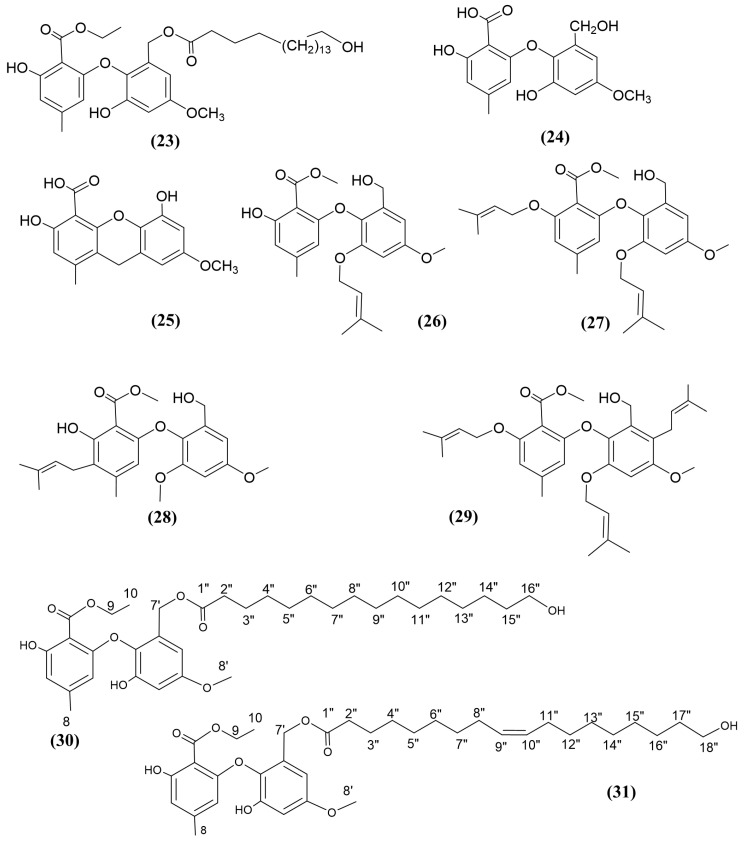
Compounds 23–31: (23) Phomaspether J, (24) Barceloneic acid A, (25) Barceloneic acid C, (26) Epicoccether A, (27) Epicoccether B, (28) Epicoccether D, (29) Epicoccether E, (30) Epicoccether F, and (31) Epicoccether G.

**Figure 6:**
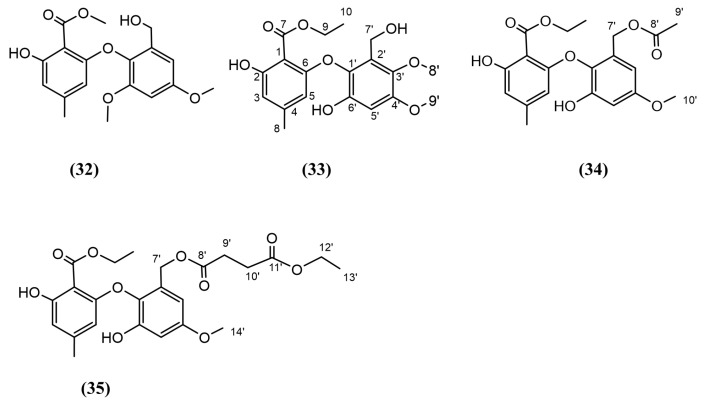
Compounds 32–35: (32) Epicoccether K, (33) Epicoccether L, (34) Epicoccether M, and (35) Epicoccether N.

Ether bonds, consisting of an oxygen atom connected to alkyl or aryl groups, are found in a wide variety of secondary metabolites spanning across all domains of life [103,104]. The leaves of the semi-mangrove plant *M. bontioides* from Leizhou Peninsula, China, was used to isolate endophytic fungus *Phoma herbarum* L28. Ethyl acetate was used to extract the fungus, which led to the purification of a novel derivative of barceloneic acid A [105,106] named phomaspether J (Figure 5, compound 23). Phomaspether J was found to display strong antifungal activity against *F. oxysporum* and *Colletotrichum musae*, with two-fold and two-fold higher antifungal activity, respectively, compared to triadimefon [106]. Closely related analogues such as barceloneic acid A and C (Figure 5, compounds 24–25) have not previously been tested for antifungal activity [105,107], which could be worth exploring. In another study, six new antifungal diphenyl ethers, epicoccethers A, B, and D-G (Figure 5, compound 26–31), were identified from the endophytic fungus *Epicoccum sorghinum* L28 isolated from *M. bontioides* [108]. Compounds 26–31 demonstrated antifungal activity against *F. oxysporum,* with potencies one-, one-, two-, one-, two-, and four-fold higher than the positive control, triadimefon, respectively. Notably, compound 31 exhibited stronger antifungal activity compared to compound 30, attributed to the extended carbon chain from C-16”, with unsaturated carbons present in between C-9” and C-10” in the long-chain moiety of compound 31. Subsequently, the same species was used to extract additional novel antifungal compounds, epicoccethers K-N (Figure 6, compounds 32–35) [109]. Among them, epicoccether N (compound 35) exhibited the strongest antifungal activity against *Penicillium italicum* and *F. graminearum*, with two-fold and three-fold higher strength, respectively, compared to the positive control, triadimefon. Furthermore, epicoccether L (compound 33) showed antifungal activity one-fold and three-fold higher for the above-mentioned plant pathogens, respectively, in comparison to triadimefon. An additional two compounds (compounds 32 and 34) also showed moderate antifungal activity for the same fungal pathogens. When comparing phomaspether J (compound 23) [106] and epicoccether N [109], both displayed strong antifungal activities against the tested pathogens. Both compounds share a similar core structure, though they differ in their long-chain moieties. However, their antifungal activities cannot be directly compared, as they were tested against different fungal pathogens. Epicoccether M (compound 34) demonstrated lower antifungal activity compared to compound 35. This could be attributed to the fact that compound 34 lacks the long-chain moiety with two ester bonds, possessing only one ester bond at C-7′, which may contribute to some degree of antifungal activity. In contrast, compound 35 exhibited stronger antifungal activity, likely due to the presence of long-chain moiety at C-7′ containing two ester bonds.

#### 3.4.5. Cyclohexenone and Cyclopentenone

Cyclohexenone and cyclopentenone are cyclic unsaturated ketones, each characterised by a carbonyl group attached to one of the carbons in the ring, along with an alkene within the ring structure [110].

**Figure 7:**
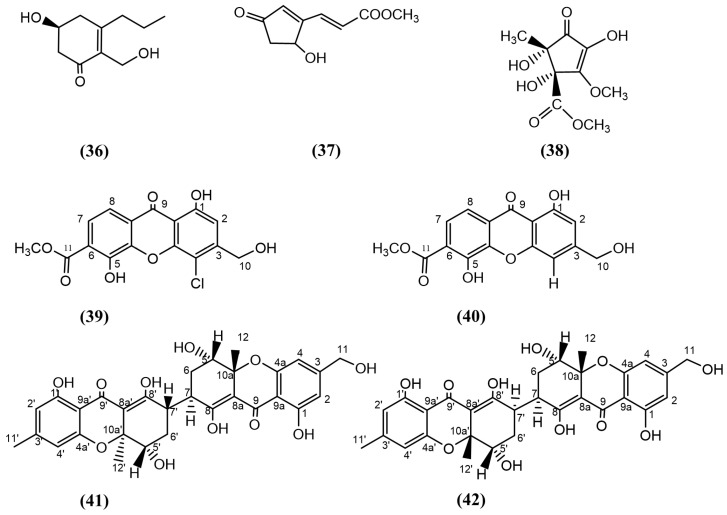
Compounds 36–42: (36) insuetone A, (37) trichodermester A, (38) (±)-(4S*,5S*)-2,4,5-trihydroxy-3-methoxy-4-methoxycarbonyl-5-methyl-2-cyclopenten1-one, (39) 4-chloro-1,5-dihydroxy-3-hydroxymethyl-6methoxycarbonyl-xanthen-9-one, (40) fischexanthone, (41) aflaxanthone A, and (42) aflaxanthone B.

A cyclohexenone called Insuetone A (Figure 7, compound 36) was isolated from a seagrass-derived endophytic fungi, *Aspergillus insuetus* SYSU6925. Insuetone A displayed antifungal activity against *F. oxysporum* and *Colletotrichum gloeosporioides*, with 50 and 100 μg/mL MIC values, respectively. The positive control, triadimefon, exhibited 100 and 50 μg/mL MIC values, respectively [111]. In another study, *Trichoderma atroviride* H548, isolated from the mangrove sediment obtained from the Estuary of Zhangjiangkou Mangrove National Nature Reserve, Fujian province, China, resulted in the isolation of a cyclopentenone acrylic acid derivative, trichodermester A (Figure 7, compound 37). This compound displayed potent antifungal activity against *Pestalotiopsis theae* [112]. In a study by [113], the researchers isolated a new cyclopentenone derivative, (±)-(4S*,5S*)-2,4,5-trihydroxy-3-methoxy-4-methoxycarbonyl-5-methyl-2-cyclopenten1-one (Figure 7, compound 38), from an *Alternaria* sp. isolated from the roots of *M. bontioides*, which displayed antifungal activities against *F. graminearum* and *Colletotrichum musae*, with MIC values of 50 and 200 μg/mL, respectively. The antifungal activity against *F. graminearum* was three-fold greater than that of the positive control, triadimefon (Table S1) [113].

#### 3.4.6. Xanthones

Xanthones are a class of organic compounds with a tricyclic scaffold characterised by a dibenzo-γ-pyrone structure. The dibenzo-γ-pyrone structure consists of two benzene rings connected by a central carbonyl and an ether group [114]. A new xanthone derivative, 4-chloro-1,5-dihydroxy-3-hydroxymethyl-6methoxycarbonyl-xanthen-9-one (Figure 6, compound 39), was isolated from the same *Alternaria* sp. that produced compound 38, mentioned in the above section (Section 3.4.5 Cyclohexenone and Cyclopentenone). This compound displayed antifungal activity against *F. graminearum* and *Colletotrichum musae* [113]. Most xanthone derivatives have previously been reported to have antifungal activities against selective fungal species [115,116,117], but the presence of chlorine in compound 39 is rare, likely enhancing the antifungal activity. The closely related analogue, fischexanthone (Figure 7, compound 40), reported by Wang, Ding [113], has a hydrogen atom instead of chlorine at C-4 in compound 39 and displayed comparatively weaker antifungal activity.

Zang, Yang [118] isolated the endophytic fungus *Aspergillus flavus* QQYZ from the mangrove plant *K. candel* collected from Huizhou in Guangdong province, China. Extraction of the fungal culture was carried out using ethyl acetate, which resulted in the isolation of two novel heterodimeric tetrahydroxanthones, aflaxanthones A and B (Figure 7, compounds 41 and 42). Aflaxanthone A exhibited significant antifungal inhibitory activity against *Colletotrichum gloeosporioides* and moderate antifungal activity against *F. oxysporum* and *Candida albicans* compared to the positive control, ketoconazole. Aflaxanthone B exhibited moderate antifungal activity against *F. oxysporum* and *Colletotrichum musae* compared to ketoconazole. These two compounds displayed varying degrees of broad-spectrum antifungal activities against different fungal species due to the differences in the 3D orientation of the hydrogen (C-7′) and the methyl group (C-12′) [118].

#### 3.4.7. Alkaloids

**Figure 8:**
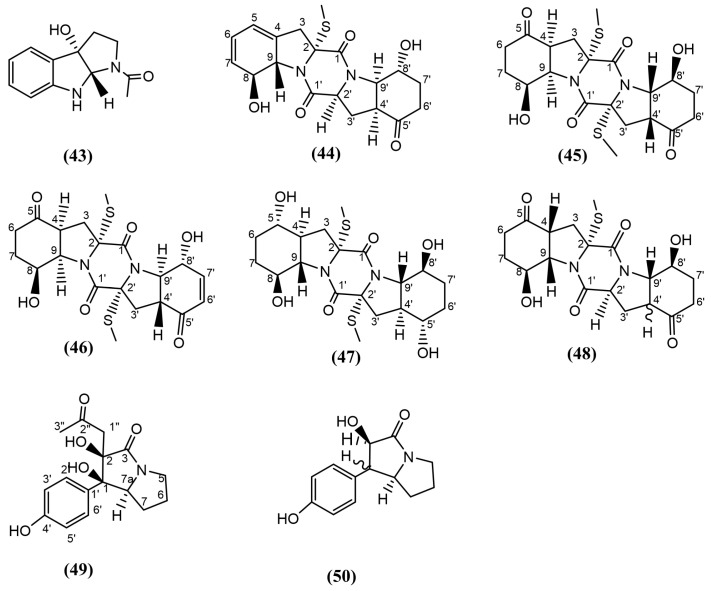
Compounds 43–50: (43) (3aS,8aS)-1-acetyl-1,2,3,3a,8,8a-hexahydropyrrolo[2,3b]indol-3a-ol, (44) penicibrocazine B, (45) penicibrocazine D, (46) penicibrocazine E, (47) penicibrocazine C, (48) penicibrocazine A, (49) brocapyrrozin A, and (50) brocapyrrozin B.

Alkaloids exists in various forms, with the majority of them consisting of a heterocyclic tertiary nitrogen in their structure [119]. The endophytic fungus *Botryosphaeria ramosa* L29, which was previously mentioned under isocoumarins (Section 3.4.3), was used to isolate (*3aS*,*8aS*)-1-acetyl-1,2,3,3a,8,8a-hexahydropyrrolo[2,3b]indol-3a-ol (Figure 8, compound 43), belonging to the class of tryptamines (a monoamine alkaloid). This compound displayed significant antifungal activity against *F. oxysporum, Penicillium italicum,* and *F. graminearum*, with MIC values of 6.24, 12.5, and 6.24 µg/mL (12-, 3-, and 18-fold stronger), respectively, compared to the known antifungal triadimefon, with MIC values of 100, 50, and 150 µg/mL, respectively [101]. In another study, three new sulphide diketopiperazine derivatives, named penicibrocazine B, D, and E (Figure 8, compounds 44, 45, and 46, respectively), were isolated from the culture extracts of *Penicillium brocae* MA-231, an endophytic fungus originating from the mangrove *Avicennia marina*. These three compounds displayed antifungal activity against the phytopathogenic species *Alternaria brassicae*, *Colletotrichum gloeosporiodes*, *F. graminearum*, and *Gaeumannomyces graminis* [120]. Sulphide diketopiperazine derivatives consist of a 6-5-6-5-6 diketopiperazine skeleton with a disulfide bridge or *S*-methyl group [120]. Penicibrocazines, which lack cyclic hexanone groups (cyclic ketone groups) on either side (C-5/5′), replaced with OH groups did not display antifungal activity. This suggests that comparing the structure of compound 46 (Figure 8), which displayed antifungal activity, with penicibrocazine C (Figure 8, compound 47), which did not possess antifungal activity [120] but possesses an S-methyl group at the 2′ position and a spatial arrangement of hydrogen atoms at positions 4, 9, 4′, and 9′ and OH moiety at position 8′, may play an important role in the observed antifungal activity. For example, compound 45 and penicibrocazine A (Figure 8, compound 48) have closely related structures. In comparison, compound 48 is composed of cyclic hexanone groups on either side at the C-5/5′ position, but one of the *S*-methyl groups at the 2′ position is replaced with a hydrogen atom and did not display any antifungal activity. In contrast, Compound 45, with two *S*-methyl groups, displayed antifungal activity [120], highlighting the role of *S*-methyl groups in antifungal activity. In 2017, the same authors utilised the same endophytic fungus, *Penicillium brocae* MA-231, and enhanced the production and diversity of secondary metabolites using the One Strain MAny Compounds (OSMAC) approach [121,122], leading to the extraction of six additional novel secondary metabolites. Among these, brocapyrrozins A and B (Figure 8, compounds 49–50), which are nitrogen-containing phydroxyphenopyrrozin alkaloids, exhibited antifungal activity against *F. oxysporum* in comparison to Zeocin (an antibacterial and antifungal used for research purposes) as the positive control. Brocapyrrozins A and B exhibited MIC values of 0.25 μg/mL and 65 μg/mL, respectively, while the positive control showed an MIC value of 0.5 μg/mL [123]. Considering the structures of brocapyrrozin B and other similar brocapyrrozins and their evaluated bioactivities, the presence of the ketone group and the OH group at position 2 on the same carbon ring of brocapyrrozin A (compound 49) is responsible for enhancing the antifungal activity in comparison to brocapyrrozin B (compound 50).

**Figure 9:**
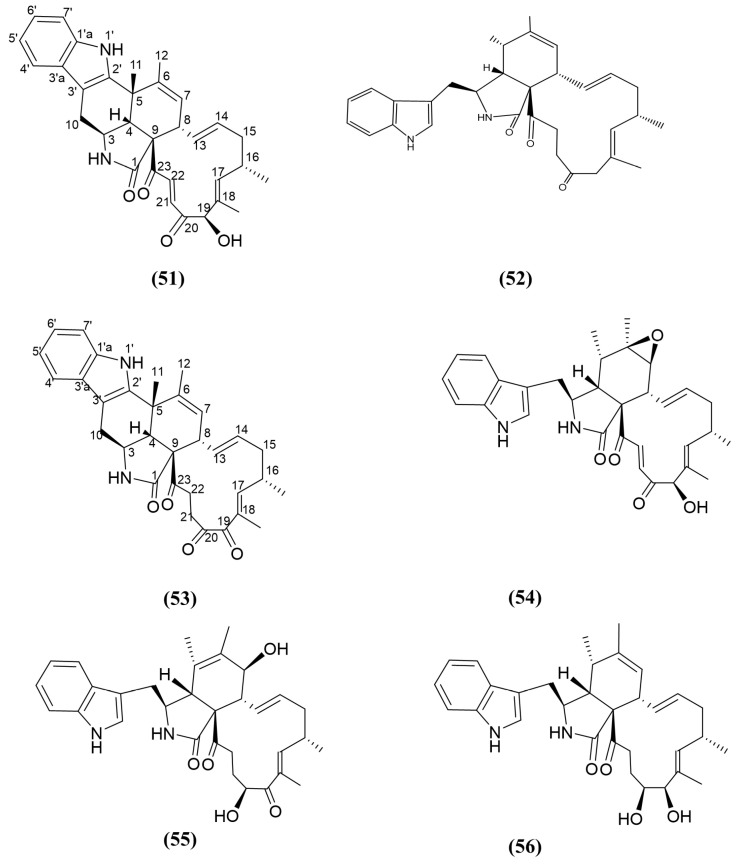
Compounds 51–56: (51) penochalasin I, (52) penochalasin J (53) penochalasin K, (54) chaetoglobosin A, (55) chaetoglobosin E, and (56) armochaetoglobosin I.

In another experiment, the endophytic fungus *Penicillium chrysogenum*, which was isolated from *M. bontioides*, led to the isolation of novel chaetoglobosins called penochalasin I and J (Figure 9, compounds 51 and 52), bearing a rare six-cyclic and common five-cyclic fused ring system, respectively. Chaetoglobosins belong to the class of cytochalasan alkaloids usually consist of a 10-(indol-3-yl) group, a macrocyclic ring, and a perhydroisoindolone moiety, with the majority of them isolated from the genus *Chaetomium*. The antifungal bioactivity of penochalasin I and J (Table S1) were assessed against the phytopathogenic fungi *Colletotrichum musae*, *Colletotrichum gloeosporioides*, *Penicillium italicum*, and *R. solani* [124]. Among them, the antifungal activity of penochalasin I was found to be mild, while the antifungal activity of penochalasin J against *Colletotrichum gloeosporioides* was two-fold greater compared to the positive control, carbendazim. According to Huang, Chen [124], when comparing the two structures of penochalasin I and J, the connection between C-5 and C-2′ reduced the antifungal activity. In the following year, another chaetoglobosin named penochalasin K (Figure 9, compound 53) bearing a rare six-cyclic fused ring system was also isolated from the same endophytic fungus previously investigated by Huang, Chen [124]. Penochalasin K displayed good antifungal activity against the same phyto-pathogenic fungi used by Huang, Chen [124], *Colletotrichum gloeosporioides* and *R. solani*, with 10-fold and 3-fold higher inhibition, respectively, compared to carbendazim [125]. However, penochalasin K (compound 53) is a compound displaying greater antifungal activity than penochalasin J, while penochalasin K possesses a C-5 and C-2′ connection; therefore, it contradicts the above statement about the strength of antifungal activity and the C-5 and C-2′ connection. When comparing penochalasin I (compound 51) [124] and penochalasin K, both compounds possess a six-cyclic fused ring system. Penochalasin K consists of a ketone at C-19 in the macrocyclic ring, whereas in penochalsin I, the C-19 position possesses a hydroxyl group. Structurally similar known analogues of penochalasin J (compound 52) such as chaetoglobosin A and E, and armochaetoglobosin I (Figure 9, compounds 54–56), isolated from the same endophyte, have been reported to have antifungal activity against *R. solani*, with a three-fold increase in antifungal activity compared to the positive control, carbendazim [124]. Further research on chaetoglobosin A revealed that it binds to the distal end of actin filaments, disrupting their dynamic assembly and disassembly. This interference leads to the inhibition of actin-mediated cell division and morphogenesis [126]. However, other similar compounds have not been investigated for their respective mode of action.

#### 3.4.8. Terpenes

**Figure 10:**
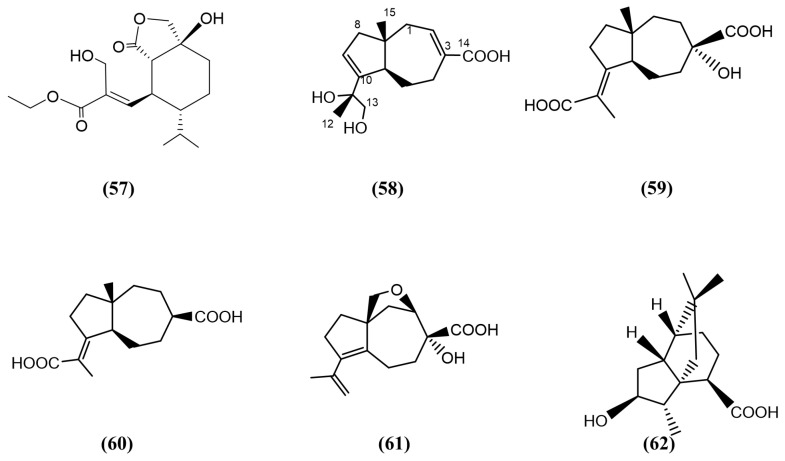
Compounds 57–62: (57) ethyl hydroheptelidate, (58–61) asperalacids A−D, and (62) 4-hydroxy-5(6)-dihydroterrecyclic acid A.

The basic structure of terpenes comprises isoprene units, with the chemical formula (C_5_H_8_)_n_. Terpenes are classified based on the number of isoprene units: monoterpenes (two isoprene units), sesquiterpenes (three isoprene units), and diterpenes (four isoprene units). Sesquiterpenes can form mono- and polycyclic rings and are further diversified with different functional groups such as alcohols, carboxylic acids, or ketones [127]. A sesquiterpene derivative, ethyl hydroheptelidate (Figure 10, compound 57) was purified from *Trichoderma harzianum* R1 isolated from *M. bontioides*, and it exhibited antifungal activity against *F. oxysporum* and *Colletotrichum gloeosporioides*, with similar potency compared to the positive control, triadimefon [128].

A seagrass species, *Enhalus acoroides*, collected from Dongzhai Port, Hainan Island, China, with necrotic leaves and normal roots, was used to isolate *Aspergillus alabamensis* SYSU-6778 [94]. Methanolic extracts of the fungus was used to isolate four new carotane sesquiterpenoid derivatives, asperalacids A−D (Figure 10, compounds 58–61) and a new tricyclic sesquiterpenoid called 4-hydroxy-5(6)-dihydroterrecyclic acid A (Figure 10, compound 62) possessing antifungal activity. Compounds 59, 60, and 62 displayed antifungal activity against *F. oxysporum*, with similar strengths to the positive control, triadimefon. Compounds 60 (with six-fold higher) and 62 (with five-fold higher) displayed strong antifungal activity, whilst other compounds displayed mild antifungal activity against *F. graminearum* in comparison to the same positive control. Only compound 62 was found to have shown weak antifungal activity against *Penicillium italicum*, while the other compounds did not display any antifungal activity for the same pathogen. Compounds 59 and 60 were closely related analogues and were the first documented examples of carotane sesquiterpenoids containing two carboxyl groups [94]. Bearing an additional functional hydroxyl group at C-3 in compound 60 enhanced the antifungal activity against *F. graminearum* by eight-fold compared to the antifungal activity of compound 59 against the same fungal pathogen [94].

#### 3.4.9. Other Fungus-Derived Compounds

**Figure 11:**
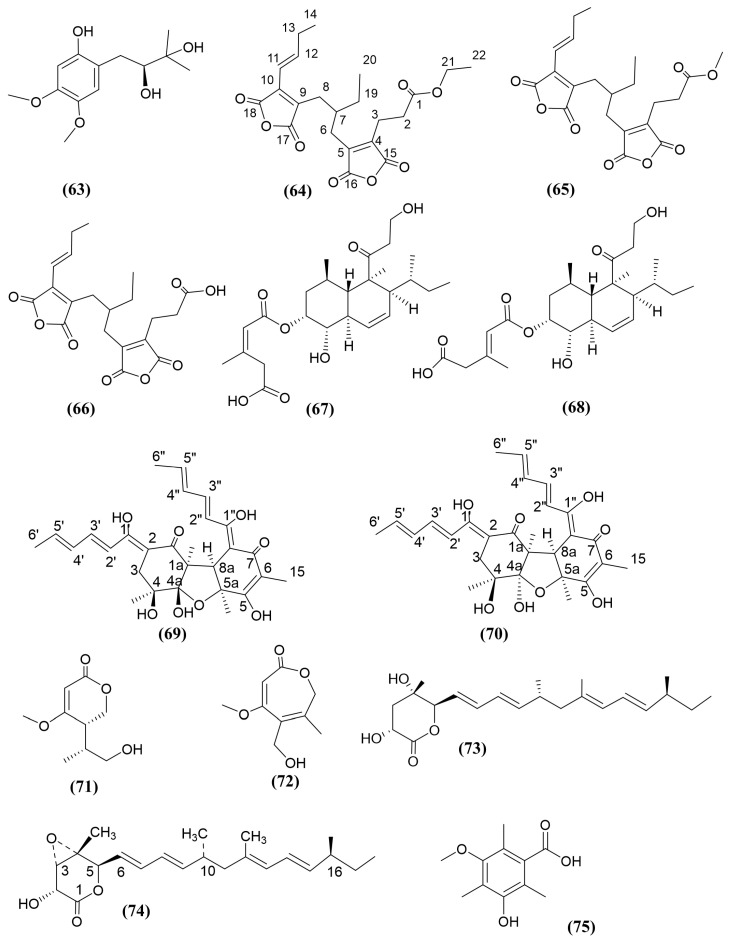
Compounds 63–75: (63) peniprenylphenol A, (64) cordyanhydride A ethyl ester, (65) cordyanhydride A methyl ester, (66) cordyanhydride A, (67) tandyukisin J, (68) trichoharzin, (69) isobisvertinol A, and (70) bisvertinol, (71) (5S, 8R)-simplicilopyrone, (72) botroxepinone, (73) nafuredin C, (74) nafuredin A, and (75) 3-hydroxy-5-methoxy-2,4,6-trimethylbenzoic acid.

A novel tetrasubstituted benzene derivative, peniprenylphenol A (Figure 11, compound 63), was isolated from the ethyl acetate extract of the Indonesian mangrove sediment-derived fungus *Penicillium chrysogenum* ZZ1151 in rice medium [129]. This compound showed antifungal activity against *C. albicans*, with a MIC value of 13 μg/mL, while the positive control, amphotericin B, exhibited an MIC value of 3.0 μg/mL [129].

A mangrove sediment-derived fungus, *Talaromyces* sp. SCSIO 41050, was used to extract a previously unreported compound, cordyanhydride A ethyl ester (Figure 11, compound 64). This compound exhibited potent antifungal activity against *Botrytis cinerea*, *F. graminearum*, *F. oxysporum*, and *R. solani*, with MIC values ranging from 12.5 to 6.25 μg/mL, which were one to two times more effective than the positive control, cycloheximide [130]. In comparing the antifungal activity of compound 64 with two other known compounds extracted from the same experiment, cordyanhydride A methyl ester and cordyanhydride A (Figure 11, compounds 65 and 66) [130], a progressive decline in antifungal potency was observed as successive methyl groups (C-21–C-22) were removed, ultimately yielding the carboxylic acid form.

In a study by Yin, Tan [96], the researchers extracted a novel antifungal tandyukusin derivative, tandyukisin J (compound 67), from the *Trichoderma lentiforme* ML-P8-2, a fungal endophyte from *Bruguiera gymnorrhiza.* This compound demonstrated antifungal activity against *C. albicans* and *Penicillium italicum*, with MIC values of 11.26 and 2.81 μg/mL, respectively, while the positive control, ketoconazole, displayed MIC values of 0.07 and 0.83 μg/mL, respectively [96]. The known compound, trichoharzin (compound 68), extracted from the same experiment and with a closely related structure, exhibited weaker antifungal activity compared to tandyukisin J. The fungal strain *Hypocrea jecorina* H8 was isolated from the mangrove sediment collected from the Mangrove National Nature Reserve, Fujian province, China. *H. jecorina* H8 was utilised to isolate the novel antifungal dimeric sorbicillinoid compound called isobisvertinol A (Figure 11, compound 69) [131]. Sorbicillinoids belongs to the class of hexaketides, which has been previously reported in fungi. These compounds feature a sorbyl side chain [132]. Isobisvertinol A was found to inhibit *Pestalotiopsis theae*, which is a tea crop pathogenic fungus with 2.5-fold higher antifungal activity than the positive control, hexaconazole, a broad-spectrum systemic triazole fungicide used in agriculture.

A known analogue structure of isobisvertinol A called bisvertinol (Figure 11, compound 70) (stereoisomer of isobisvertinol A) with a 3D arrangement variation in the OH group at C-4a, isolated from the same experiment, did not exhibit antifungal activity against the same pathogen [131]. The toxicity evaluation, based on zebrafish embryo mortality, revealed that a 10 μM concentration of compound 69 caused less than 50% mortality, regardless of the treatment duration. However, the compound demonstrated a more pronounced effect on zebrafish mortality and malformation at both 0.625 μM after 24 h and 10 μM after 72 h. Toxicity evaluation studies have not been conducted for compound 70 [131]. According to Hu, Wu [97], apart from the four chromone derivatives described in Section 3.4.2, (5*S*, 8*R*)-simplicilopyrone (a new δ-lactone) (Figure 11, compound 71) and botroxepinone (a new 2-(7*H*)-oxepinone) (Figure 11 compound 72) exhibited antifungal activities against *F. oxysporum*, *Colletotrichum musae*, and *F. graminearum* [97].

In a study by Zhao and co-authors, isolated *Trichoderma harzianum* D13 from the roots of the mangrove *Excoecaria agallocha* Linn, which produced the newly identified polyketide derivative nafuredin C (Figure 11, compound 73), displayed antifungal activity against *Magnaporthe oryzae*, causing rice blast disease [133]. Nafuredin C contains a lactone moiety with two hydroxy groups and a methylated olefinic side chain. Similar compounds were reported by Shiomi, Ui [134], which exhibited cytotoxic and antihelmintic activities. In addition to this, a known compound, nafuredin A (Figure 11, compound 74), containing one hydroxy group attached to a lactone moiety at C-2, showed less antifungal activity compared to nafuredin C [133].

Fungal endophyte *Phoma* sp. SYSU-SK-7 was isolated from a healthy branch of the mangrove species *K. candel* from the Mangrove Nature Reserve in Guangxi province, China. Out of the five new polyketide compounds isolated, 3-hydroxy-5-methoxy-2,4,6-trimethylbenzoic acid (Figure 11, compound 75) demonstrated anticandidal activity and antibacterial activity against *Bacillus subtilis* [135].

In a study by Gashgari, Gherbawy [59], the authors investigated the stems and roots of seven medicinal plants (*Achillea fragrantissima*, *Neurospora retusa*, *Alhagi graecorum*, *Citrullus colocynthis*, *Cressa cretica*, *Artemisia sieberi*, and *Tamarix nilotica*) collected from the salt marshes of Al-Gouf Governorate, Kingdom of Saudi Arabia. All seven medicinal plants hosted one or more species of endophytes with differences in the frequencies of isolation. *Mycelia sterilia* and *Penicillium chrysogenum* were the most frequently isolated endophytes, whereas *Penicillium chrysogenum*, *Emericella crustaceum*, *Aspergillus sydowii*, and *Fusarium brachygibbosum* showed the highest level of antifungal bioactivity against all three plant pathogenic fungi tested, namely, *F. oxysporum*, *F. solani*, and *Alternaria* sp. [59]. It is interesting to note that *Fusarium brachygibbosum* produced antifungal compounds against pathogenic *F. oxysporum* and *F. solani*, which exemplifies inhibiting members of their own genus. In another study, three salt marsh plants, *Phragmites australis* (Poaceae), *Suaeda salsa* (Chenopodiaceae), and *Arundo donax* (Poaceae), were used to study fungi present in the rhizosphere, with the aim of isolating safe biofertilisers and biofungicides for biosaline agriculture in Jiangsu province of China [42]. The mycoparasitic fungi *Trichoderma asperelloides* and *Trichoderma arenarium* (phylum ascomycota, family *Hypocreaceae*) showed weak antagonistic activity against five phytopathogenic fungi, including *Alternaria* cf. *alternata*, *Rhizotonia solani*, *Macrophoma* sp. *Fusarium odoratissimum*, and *Pestalotiopsis fici*, based on dual confrontation assays [42]; however, there was no record of novel antifungal bioactive compounds isolated from this study.

### 3.5. Antifungals from Endophytic and Rhizospheric Bacteria

#### 3.5.1. Macrolides

**Figure 12:**
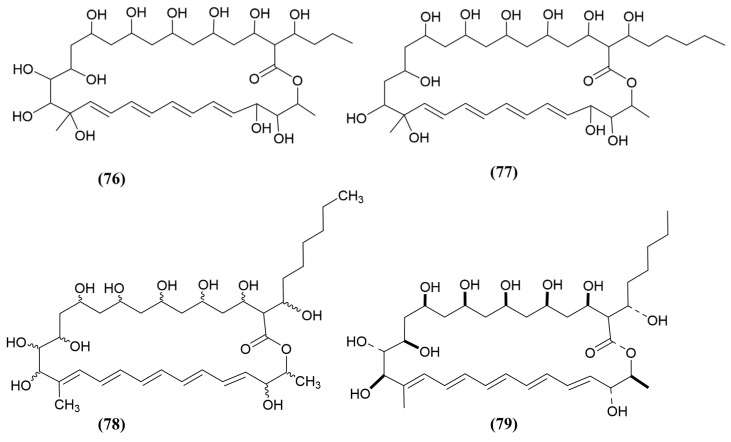
Compounds 76–79: (76) antifungalmycin B, (77) antifungalmycin E, (78) fungichromin B (6′-methyl-fungichromin), and (79) fungichromin.

A mangrove-derived *Streptomyces hiroshimensis* GXIMD 06359 was used to isolate two new antifungal polyene macrolides, antifungalmycin B and E (Figure 12, compounds 76 and 77), with MIC values of 16 and 32 μg/mL, respectively, against *Talaromyces marneffei.* In comparison, the positive controls, fluconazole and amphotericin B, exhibited MIC values of 16 and 0.5 μg/mL, respectively [136]. The research team further investigated the mode of action of antifungalmycin B, which was found to disrupt the cell membrane, inducing mitochondrial dysfunction and blocking the respiratory chain. This occurred through a reduction in ATPase levels and the subsequent inactivation of succinate dehydrogenase and malate dehydrogenase, ultimately leading to cell death [136].

In a study by Zeng, Huang [137], the authors isolated a novel compound, 6′-methyl-fungichromin, named fungichromin B (Figure 12, compound 78), a derivative of a conjugate pentane macrolide, from *Streptomyces albogriseolus* HA10002, isolated from mangrove sediment from the Dongzhaigang Mangrove Reserve in Hainan, China. Filter paper discs containing 0.10 μg of fungichromin B displayed antagonism against *Saccharomyces cerevisiae*, *F. oxysporum*, and *A. niger* [137]. However, the authors did not report a positive control in this experiment and could not find any further studies related to the mode of action of fungichromin B. Fungichromin (Figure 12, compound 79) is an analogue of fungichromin B (compound 78), derived from *Streptomyces padanus.* Compound 79 lacks one carbon compared to compound 78 in the long carbon chain, and displayed antifungal activity against *R. solani* [138]. In the study, *R. solani* treated with culture filtrates of *S. padanus* strain showed signs of necrosis and fractures when examined using scanning electron microscopy [138]. However, further studies on fungichromin displayed improved downregulation of some genes related to biofilm formation, such as *ALS1*, *ALS3*, *HWP1*, *EFG1*, *HYR1*, *CPH1*, and *BCR1*, followed by inhibition of biofilm formation in *C. albicans* at higher concentrations compared to the positive controls, fluconazole and amphotericin B. Additionally, fungichromin induced apoptosis of *C. albicans* cells in a dose-dependent manner [139].

**Figure 13:**
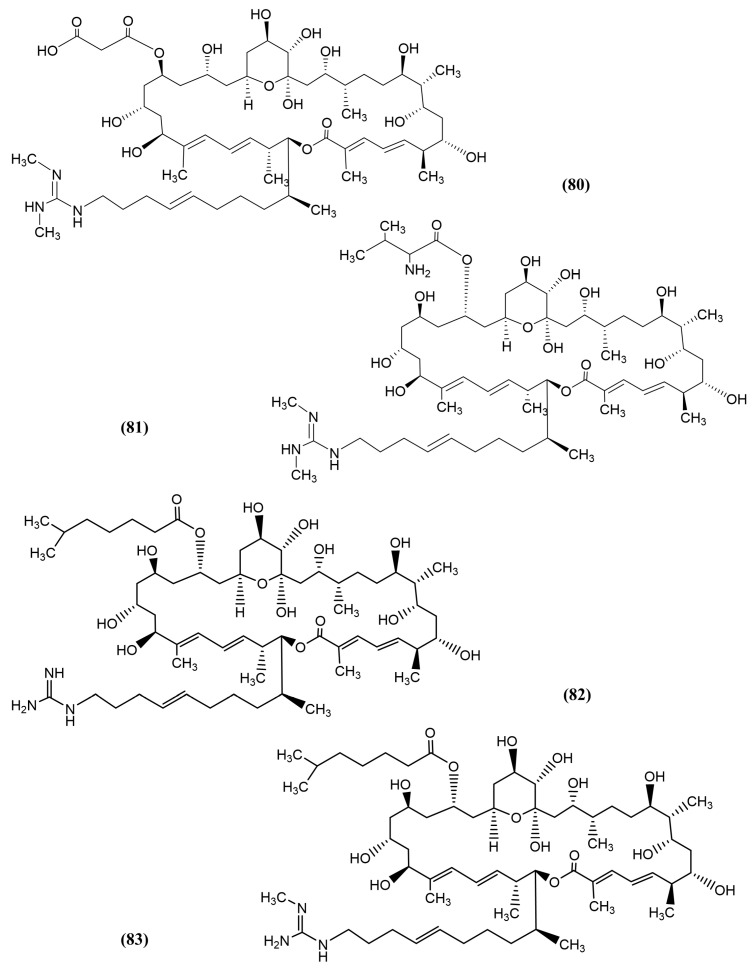
Compounds 80–83: (80) 25-malonyl demalonylazalomycin F5a monoester, (81) 23-valine demalonylazalomycin F5a ester, (82) 23-(6-methyl)heptanoic acid demalonylazalomycins F3a ester, and (83) azalomycin F analogue 4 (F4a ester).

**Figure 14:**
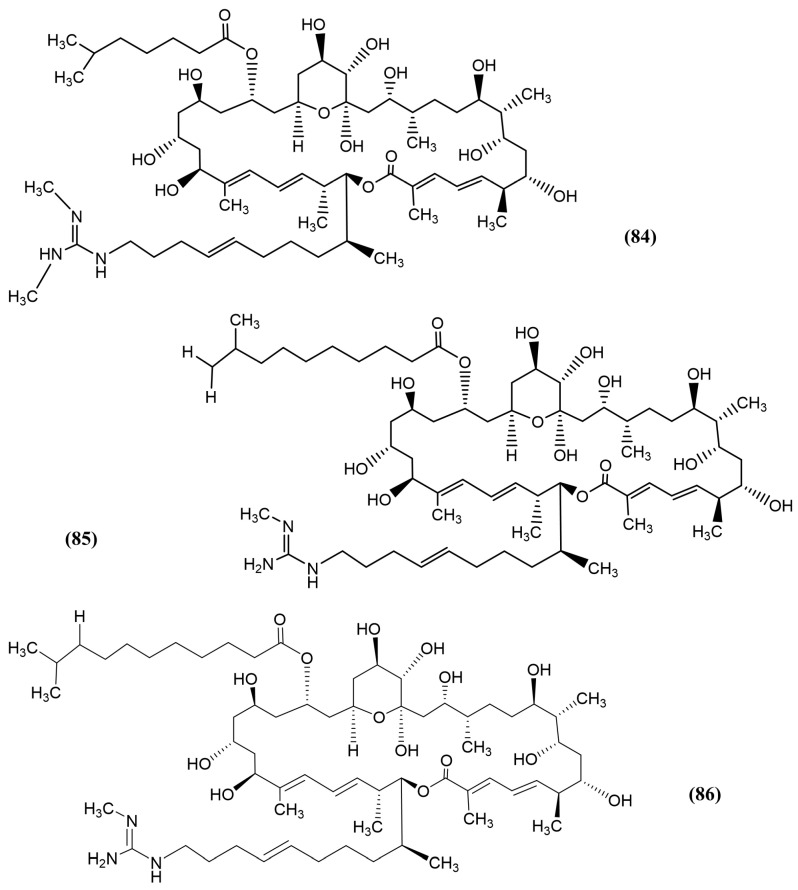
Compounds 84–86: (84) azalomycin F analogue 5 (F5a ester), (85) 23-(9-methyl)decanoic acid demalonylazalomycin F4a ester, and (86) 23-(10-methyl)undecanoic acid demalony lazalomycin F4a ester.

In another study, *Streptomyces* strain 211726 was isolated from the mangrove soil rhizosphere *Heritiera globosa* by Yuan et al. in China. Seven new azalomycin F analogues (macrolides) were identified, namely, 25-malonyl demalonylazalomycin F5a monoester (Figure 13, compound 80), 23-valine demalonylazalomycin F5a ester (Figure 13, compound 81), 23-(6-methyl)heptanoic acid demalonylazalomycins F3a ester (Figure 13, compound 82), F4a ester (Figure 13, compound 83), F5a ester (Figure 14, compound 84), 23-(9-methyl)decanoic acid demalonylazalomycin F4a ester (Figure 14, compound 85), and 23-(10-methyl)undecanoic acid demalonylazalomycin F4a ester (Figure 14, compound 86). All seven compounds showed significant antifungal activity, in which F4a ester (compound 83) and F5a ester (compound 84) demonstrated similar activity compared to the positive control, amphotericin B [140]. Most of these azalomycins are esterified compounds that exhibited antifungal activity. Niphimycin, an analogue of azalomycin F5a, exhibited potent antifungal activity by synergistically damaging the plasma membrane and inducing oxidative stress [141].

#### 3.5.2. Other Bacterial-Derived Compounds

**Figure 15:**
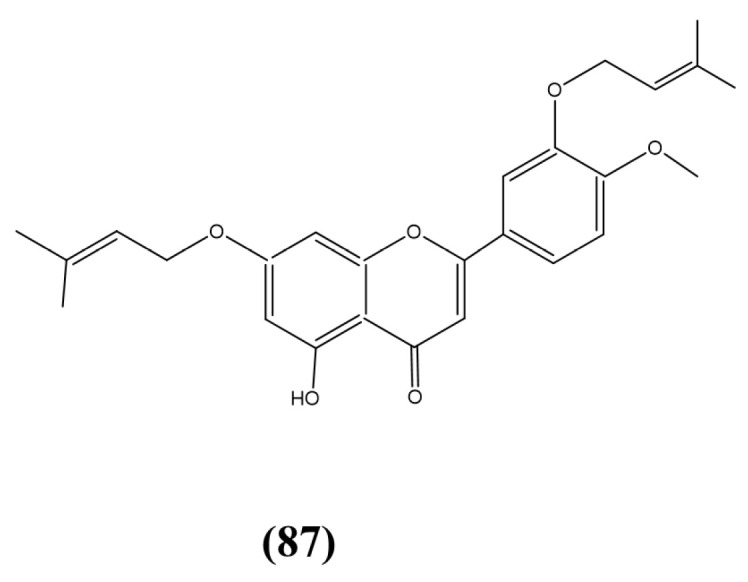
Compound 87: (87) 7,30-di-(g,g-dimethylallyloxy)-5-hydroxy-40-methoxyflavone.

A semi-mangrove plant, *M. bontioides*, resulted in the isolation of the endophyte *Streptomyces* sp. strain MA-12 [142]. This species produced a novel 7,30-di-(*g,g*-dimethylallyloxy)-5-hydroxy-40-methoxyflavone (Figure 15, compound 87), a di-*O*-prenylated flavone that displayed antifungal activities against *Colletotrichum musae*, *Gibberella zeae* (Schweinitz) Petch, and *Penicillium citrinum* Thom. The presence of a prenyl group in flavones correlated to the observed antimicrobial activity [143]. However, possessing a prenyl group is not the only qualification of antifungal activity [143], as for compound 87 the prenyl group is connected through an oxygen atom (prenyloxy), which is uncommon.

In the coastal areas of Jeddah, Saudi Arabia, the seagrass *Halodule uninervis* and its associated bacterial endophytes have been evaluated for antagonistic potential against fungal pathogens. Among the 162 rhizospheric and endophytic bacteria isolated from the soil, roots, and leaves of *H. uninervis*, 19 bacterial isolates (11.7%) displayed antifungal bioactivity against four plant pathogenic fungi, including *Pythium ultimum*, *R. solani, Phytophthora capsici*, and *pyricularia oryzae*. Out of the 162 endophytic and rhizospheric bacterial isolates, *Sulfitobacter dubius* KMM 3554, *Staphylococcus epidermidis* ATCC 14990, *Jeotgalicoccus aerolatus* MPA-33, and *Staphylococcus hominis* subsp. *novobiosepticus* GTC 1228 displayed broad-spectrum antifungal activities, with an 8–12 mm zone of inhibition in in vitro antagonistic assays; however, there were no records of antifungal compounds isolated from this study [63].

## 4. Conclusions

The diversity and the prospective bioactivities of microorganisms that coastal vegetation harbours offers a promising source of antifungal natural products. While mangrove fungal EMs and RMs have been extensively studied, research on other coastal plants remains limited. Among the plant-associated fungi in the coastal vegetation, *Penicillium* species are the most common, followed by *Aspergillus* sp., *Fusarium* sp., *Alternaria* sp., and *Cladosporium* sp. Common plant-associated bacteria include Pseudomonadota, Bacillota, and Actinomycetota.

Over the past 10 years, a total of 65 novel antifungal compounds have been discovered from coastal vegetation-associated EMs and RMs. Of these, 54 compounds were derived from fungi and 11 from bacteria belonging to Actinomycetes. In comparison to endophytic and rhizospheric fungus-derived antifungals, bacterium-derived antifungals are rarely reported. Among bacterial EMs and RMs, the class Actinomycetia stands out as the most promising source of antifungal natural products, serving as a reservoir for numerous potential antifungal candidates with pharmaceutical applications [65].

Almost all fungal-derived antifungals have shown effectiveness against phytopathogens, such as *Fusarium* sp., *G. graminis*, and *Colletotrichum* sp. In contrast, most bacterial endophytic antifungals have reported activity against *Candida albicans*. Therefore, it is crucial to screen endophytic fungus-derived antifungals for efficacy against human fungal pathogens.

This review includes five novel naphthalene derivatives, five chromone derivatives, five isocoumarines, eleven ether compounds, three xanthones, eight alkaloids, six terpenes, three cyclohexanone and cyclopentenones, and eight other compounds from fungal EMs and RMs. Bacterial partners contributed ten macrolides and one flavone displaying antifungal activities.

Overall, these novel compounds have not undergone toxicological evaluations, except for one instance. Therefore, it is recommended to include toxicological studies alongside the assessment of antifungal bioactivities for novel compounds.

Mangroves have emerged as the most prominent host plants for the discovery of novel antifungal compounds compared to other coastal plants. The semi-mangrove plant *M. bontioides* has been identified as a rich source of fungal endophytes that have yielded numerous novel antifungals to date. Coastal plant communities such as mangroves, sand dunes, salt marshes, and seagrasses offer untapped potential for discovering antifungal natural products, though they remain underexplored, providing opportunities for future research.

Finally, it is worth mentioning that the mode of action is not explored in the majority of studies presenting novel compounds, with only a few being investigated further in this regard.

## Figures and Tables

**Table 1:** Diversity of endophytes and/or rhizospheric fungi associated with coastal vegetation.

Coastal Ecosystem/Vegetation	Plant Species	Abundant Endophytic and Rhizospheric Microbes	Reference
Korean coastal sand dune plant species	*Elymus mollis*, *Argusia sibirica*, *Raphanus sativus*, *Lithospermum zollingeri*, *Salsola collina*, *Calystegia soldanella*, *Zoysia macrostachya*, and *Z. sinica*	**Division**—Ascomycota 96% **Order**—Eurotiales (70.10%), Hypocreales (9.28%), Capnodiales (3.09%), and Helotiales (3.09%) **Genera**—*Penicillium* (59.18%) and *Fusarium* sp. (5.10%)	[60]
Marine cliff grass of North Atlantic coast of Spain	Roots of *Festuca rubra* sub sp. *pruinose*	**Division**—Ascomycota 96% **Order**—Pleosporales (23%), Hypocreales (18%), Eurotiales (10%), Xylariales, and Helotiales **Genera**—*Fusarium* sp., *Diaporthe* sp., *Helotiales* sp., and *Penicillium* sp. (71.8% were halotolerant)	[58]
Salt marsh vegetation in North Saudi Arabia	*Alhagi graecorum*, *Cressa cretica*, *Citrullus colocynthis*, *Tamarix nilotica*, *Achillea fragrantissima*, *Artemisia sieberi*, and *Neurospora retusa*	**Division**—Ascomycota (58.54%) and Deuteromycota (39.27%) **Species—***Penicillium chrysogenum* (CF—98.57%) and *Mycelia sterilia* (CF—92.86%)	[59]

CF—colonisation frequency.

**Table 2:** Diversity of endophytic and/or rhizospheric bacteria associated with coastal vegetation.

Coastal Ecosystem/Vegetation	Plant sp.	Abundant Endophytic and Rhizospheric Microbes	Reference
Salt marsh ecosystems of Gujarat Coast, India	*Salicornia brachiata*	**Phyla**—Bacillota, Pseudomonadota, and Actinomycetota **Genera**—*Bacillus, Paenibacillus, Enterococcus, Nitratireductor, Pseudomonas, Salinicola, Rhodococcus, Streptomyces, Nocardiopsis, Micromonospora Isoptericola,* and *Nocardiopsis*	[64]
Mangrove from Central Red Sea, Saudi Arabia	*Avicennia marina* propagules	**Phyla**—Pseudomonadota, Bacillota, Actinomycetota, and Bacteroidota **Genera***—Acinetobacter, Staphylococcus, Corynebacterium,* and *Micrococcus*	[62]
Mangrove from Jeddah, Saudi Arabia	*Salsola imbricata*, *Avicennia marina*, *Avicennia germinans*, *Halopeplis perfoliata*, *Halocnemum strobilaceum*, *Cyperus conglomeratus*, and *Zygophyllum qatarense*	**Phyla**—Pseudomonadota, Bacillota, and Actinomycetota **Genera**—*Erwinia*, *Vibrio*, *Psychrobacter*, *Aidingimonas*, *Marinobacter*, *Chromohalobacter*, *Bacillus,* and *Isoptericola*	[63]
Native coastal salt marshes in Jiangsu, China.	**Plants used for culture—independent study** *Phragmites australis*, *Sesbania cannabina*, *Chrysanthemum indicum*, *Metaplexis japonica*, *Suaeda glauca*, *Lycium linn*, and *Spartina alterniflora*	**Culture—independent endophytic bacterial taxa** **Phyla**—Pseudomonadota, Actinomycetota, Bacillota, and Bacteroidota **Orders**—Acidimicrobiales, Streptomycetales, and Micrococcales **Genera***—Streptomyces*, *Mycobacterium*, *Arthrobacter*, *Micrococcus*, *Micromonospora*, *Herbiconiux*, and *Actinoplanes*	[73]
	**Plants used for culture—dependent study** *Tamarix chinensis*, *Dendranthema indicum*, *Salicornia europaea*, *Sesbania cannabina*, *Spartina alterniflora*, and *Suaeda glauca*	**Culture—dependent endophytic bacteria** grown on selective media **Phylum**—Actinomycetota **Order**—Streptomycetales, Pseudonocardiales, and Micrococcales **Genera***—Streptomyces* (60%), *Saccharopolyspora*, *Pseudonocardia*, *Dietzia*, *Kineococcus*, *Citriococcus*, and *Janibacter*	
Mangrove plants collected from Beilun Estuary National Nature Reserve, China	*Avicennia marina*, *Aegiceras corniculatum*, *Kandelia obovota*, *Bruguiera gymnorrhiza*, and *Thespesia populnea*	**Phylum**—Actinomycetota **Genera***—Streptomyces*, *Curtobacterium*, *Mycobacterium*, *Brevibacterium*, *Micrococcus*, *Kocuria*, *Nocardioides*, *Kineococcus*, *Nocardia*, and *Microbacterium*	[72]

## Data Availability

No new data were created or analysed in this study. Data sharing is not applicable to this article.

## Supplementary Materials

The following supporting information can be downloaded at: https://www.mdpi.com/article/10.3390/jox15010032/s1, Table S1: Antifungal compounds isolated from endophytic and/or rhizospheric fungi associated with coastal vegetation; Table S2: Antifungal compounds discovered from endophytic and rhizospheric bacteria including Actinomycetes associated with coastal vegetation.
