# Supplementary material for: Antifungal Natural Products Originating from Endophytic and Rhizospheric Microbes Isolated from Coastal Vegetation
Source: J Xenobiot. 2025 Feb 17;15(1):32. doi: 10.3390/jox15010032 (PMC11856389; doi:10.3390/jox15010032)
Supplement: Supplementary file 1 [file jox-15-00032-s001.zip › jox-3390668-supplementary.pdf]

Journal of *Xenobiotics*

MDPI

Table S1. Antifungal compounds isolated from endophytic and/or rhizospheric fungi associated with coastal vegetation.

**Compound Number and Name**	**Associated Biological Activity[¥]**	**Antifungal Producing Endophytic and/or Rhizospheric Species**	**Host Plant/ Source**	**Location**	**Reference**
**Naphthalene derivatives**					
**(1) Dalesconoside A**<br><br>MF*: $C_{17}H_{20}O_7$<br>MW[#]: 336.1289 g/mol	Antifungal activity against *C. albicans* was observed, with an MIC of 25 µg/mL (positive control was amphotericin B, with an MIC value of 0.78 µg/mL)	*Daldinia eschscholzii* MCZ-18	Healthy branch of *Ceriops tagal*	Dongzhaigang Mangrove Garden on Hainan Island, China	[87]
**(3) Cladonaphchrom A**<br>MF: $C_{22}H_{22}O_4$<br>MW: 350.1443 g/mol	Antifungal activities against *Alternaria brassicicola*, *Phytophthora parasitica* var. nicotianae, *Colletotrichum capsici*, *Bipolaris oryzae*, *Diaporthe medusaea* Nitschke, and *Ceratocystis paradoxa* Moreau were observed, with MIC values of 50, 50, 25, 100, 50, and 50 µg/mL respectively (positive control was prochloraz, with MIC values	*Cladosporium* sp. JJM22	*Ceriops tagal*	South China Sea	[88]

**Compound Number and Name**	**Associated Biological Activity¥**	**Antifungal Producing Endophytic and/or Rhizospheric Species**	**Host Plant/ Source**	**Location**	**Reference**
	of 12.5, 50, 12.5, 50, 50, and 25 μg/mL, respectively).				
**(4) Cladonaphchrom B**<br><br>MF: $C_{22}H_{22}O_4$<br>MW: 350.1448 g/mol	Antifungal activities against *A. brassicicola*, *Phytophthora parasitica* var.nicotianae, *Colletotrichum capsici*, *B. oryzae*, *D. medusaea* Nitschke, and *Ceratocystis paradoxa* Moreau were observed, with MIC values of 25, 50, 25, >200, 100, and 50 μg/mL respectively (positive control was prochloraz, with MIC values of 12.5, 50, 12.5, 50, 50, and 25 μg/mL, respectively).	*Cladosporium sp.* JJM22	*Ceriops tagal*	South China Sea	[88]
**(5) Guignardin B**<br><br>MF: $C_{20}H_{14}O_5$<br>MW: 334.0763 g/mol	Antifungal activities against *Fusarium* sp. and *Aspergillus niger* were observed, with 6 and 7 mm inhibition zones at 25 μg/disc content, respectively (positive control carbendazim showed 18 and 10 mm zones of inhibition at the same amount of disc content, respectively).	*Guignardia* sp. KcF8	Fresh healthy fruits of mangrove plant *Kandelia candel*	Guangdong province, China	[90]

**Compound Number and Name**	**Associated Biological Activity**[¥]	**Antifungal Producing Endophytic and/or Rhizospheric Species**	**Host Plant/ Source**	**Location**	**Reference**
**(9)** **(3S)-3,8-dihydroxy-6,7-dimethyl-α-tetralone** MF: $C_{12}H_{14}O_3$ MW: 206.0943 g/mol	Antifungal activity against *Microsporum gypseum* was observed, with an MIC value of approximately 200 mg/mL. No positive control	*Daldinia eschscholtzii* PSU-STD57	A leaf of the mangrove plant *Bruguiera gymnorrhiza* (L.)	Suratthani province, Thailand	[92]
**Chromone derivatives**					
**(10) 5-hydroxy-3-hydroxymethyl-7-methoxy-2-methyl-4-chromanone** MF: $C_{12}H_{14}O_5$ MW: 238.0916	Antifungal activity against *Aspergillus alabamensis* was observed, with an MIC value of 100 µg/mL (positive control was triadimefon, with an MIC value of 100 µg/mL).	Mixed culture of *Aspergillus alabamensis* SYSU-6778 and co-isolated fungus *Aspergillus fumigatiaffinis* SYSU-6786	*Aspergillus alabamensis* SYSU-6778 infected necrotic leaves and *Aspergillus fumigatiaffinis* SYSU-6786 isolated from normal roots of *Enhalus acoroides* infected roots of *Enhalus acoroides*	Hainan Island, the People's Republic of China	[95]
**(11) 5-hydroxy-3-(3′R, 5′S)-3′-hydroxy-**	Antifungal activity against *C. albicans* was observed, with an MIC value of 15.25 µg/mL	*Trichoderma lentiforme* ML-P8-2	Fresh leaf of *B. gymnorrhiza*	Dongzhaigan g	[96]

**Compound Number and Name**	**Associated Biological Activity¥**	**Antifungal Producing Endophytic and/or Rhizospheric Species**	**Host Plant/ Source**	**Location**	**Reference**
**2′-oxotetrahydrofuran-5′-yl)-7-methoxy-2-methyl-4H-chromen-4-one**<br>MF: $C_{15}H_{14}O_7$<br>MW: 305.0732	(positive control was ketoconazole, with an MIC value of 0.07 µg/mL).			National Nature Reserve in Hainan province, China	
**(12)**<br>**5-hydroxy-2, 3-dihydroxymethyl-7-methoxychromone**<br>MF: $C_{12}H_{12}O_6$<br>MW: 252.0713 g/mol	Antifungal activities against *Fusarium oxysporum*, *Colletotrichum musae*, *Penicillium italicum Wehme*, and *Fusarium graminearum* were observed, with MIC values of 50, 200, 6.25, and 100 µg/mL, respectively (positive control was triadimefon, with MIC values of 100, 80,50, and 150 µg/mL, respectively).	*Botryosphaeria ramosa* L29	Leaf of *Myoporum bontioides*	Leizhou Peninsula, China	[97]
**(13)**<br>**5-hydroxy-3-acetoxymethyl-2-methyl-7-methoxychromone**<br>MF: $C_{14}H_{14}O_6$<br>MW: 278.0871 g/mol	Antifungal activities against *F. oxysporum*, *Colletotrichum musae*, *Penicillium italicum*, and *F. graminearum* were observed, with MIC values of 50, 100, 100, and 50 µg/mL, respectively (positive control was triadimefon, with MIC values of 100, 80,50, and 150 µg/mL, respectively).	*Botryosphaeria ramosa* L29	Leaf of *M. bontioides*	Leizhou Peninsula, China	[97]

Compound Number and Name	Associated Biological Activity¥	Antifungal Producing Endophytic and/or Rhizospheric Species	Host Plant/ Source	Location	Reference
**(14)**<br>**5, 7- dihydroxy-3-hydroxymethyl-2-methylchromone**<br><br>MF: $C_{11}H_{10}O_5$<br>MW: 222.0599 g/mol	Antifungal activities against *F. oxysporum*, *Colletotrichum musae*, *Penicillium italicum*, and *F. graminearum* were observed, with MIC values of 6.25, 12.5, 12.5, and 100 μg/mL, respectively (positive control was triadimefon, with MIC values of 100, 80, 50, and 150 μg/mL, respectively).	*Botryosphaeria ramosa* L29	Leaf of *M. bontioides*	Leizhou Peninsula, China	[97]
**Isocoumarines**					
**(15)**<br>**8-hydroxy-3-hydroxymethyl-6-methoxy-7-methylisocoumarin**<br><br>MF: $C_{12}H_{12}O_5$<br>MW: 236.0763 g/mol	Antifungal activities against *F. oxysporum*, *Colletotrichum musae*, *Penicillium italicum*, and *F. graminearum* were observed, with MIC values of 100, 200, 12.5, and 100 μg/mL, respectively (positive control was triadimefon, with MIC values of 100, 80,50, and 150 μg/mL, respectively).	*Botryosphaeria ramosa* L29	Leaf of *M. bontioides*	Leizhou Peninsula, China	[97]
**(19) Botryospyrone A**<br><br>MF: $C_{11}H_{10}O_5$<br>MW: 222.06 g/mol	Antifungal activity against *F. oxysporum* was observed, with an MIC value of 25 μg/mL (positive control was triadimefon, with an MIC value of 100 μg/mL).	*Botryosphaeria ramosa L29*	Leaf of *M. bontioides*	Leizhou Peninsula, China	[101]

Compound Number and Name	Associated Biological Activity¥	Antifungal Producing Endophytic and/or Rhizospheric Species	Host Plant/ Source	Location	Reference
**(20) Botryospyrone B**<br><br>MF: $C_{12}H_{12}O_5$<br>MW: 236.07 g/mol	Antifungal activities against *F. oxysporum*, *Penicillium italicum*, and *F. graminearum* were observed, with MIC values of 24.98, 49.98, and 49.98 µg/mL, respectively (positive control was triadimefon, with MIC values of 100, 50, and 150 µg/mL, respectively).	*Botryosphaeria ramosa L29*	Leaf of *M. bontioides*	Leizhou Peninsula, China	[101]
**(21) Botryospyrone C**<br><br>MF: $C_{11}H_{12}O_5$<br>MW: 224.07 g/mol	Antifungal activities against *F. oxysporum, Penicillium italicum,* and *F. graminearum* were observed, with MIC values of 49.97, >201.67, and 49.97 µg/mL, respectively (positive control was triadimefon, with MIC values of 100, 50, and 150 µg/mL, respectively).	*Botryosphaeria ramosa L29*	Leaf of *M. bontioides*	Leizhou Peninsula, China	[101]
**(22)** Dichlorodiaportinolide (1)<br><br>MF: $C_{16}H_{14}C_{12}O_7$<br>MW: 388.0184 g/mol	Antifungal activities against *Colletotrichum musae* and *Rhizoctonia solani* Kühn were observed, with MIC values of 25 and 6.25 µg/mL, respectively (positive control	*Trichoderma sp.* 09	A semi-mangrove plant *M. bontioides*	Leizhou Peninsula, China	[102]

Compound Number and Name	Associated Biological Activity¥	Antifungal Producing Endophytic and/or Rhizospheric Species	Host Plant/ Source	Location	Reference
	was carbendazim, with MIC values of 6.25 μg/mL for both pathogens).				
**Ether compounds**					
**(23) Phomaspether J**<br><br>MF: $C_{36}H_{54}O_9$<br>MW: 630.3763 g/mol	Antifungal activities against *F. oxysporum* and *Colletotrichum musae* were observed, with MIC values of 50 and 100 μg/mL, respectively (positive control was triadimefon, with MIC values of 100 and 80 μg/mL, respectively).	*Phoma herbarum* L28	Leaves of *M. bontioides*	Leizhou Peninsula, China	[106]
**(26) Epicoccether A**<br><br>MF: $C_{22}H_{26}O_7$<br>MW: 402.1602 g/mol	Antifungal activities against *F. oxysporum* and *Colletotrichum musae* were observed, with MIC values of 100 and 200 μg/mL, respectively (positive control was triadimefon, with MIC values of 100 and 80 μg/mL, respectively).	*Epicoccum sorghinum* L28	*M. bontioides*	Leizhou Peninsula, China	[108]
**(27) Epicoccether B**<br><br>MF: $C_{27}H_{35}O_7$<br>MW: 470.2384 g/mol	Antifungal activities against *F. oxysporum* and *Colletotrichum musae* were observed, with MIC values of 100 and >200 μg/mL,	*Epicoccum sorghinum* L28	*M. bontioides*	Leizhou Peninsula, China	[108]

**Compound Number and Name**	**Associated Biological Activity¥**	**Antifungal Producing Endophytic and/or Rhizospheric Species**	**Host Plant/ Source**	**Location**	**Reference**
	respectively (positive control was triadimefon, with MIC values of 100 and 80 μg/mL, respectively).				
**(28) Epicoccether D**<br><br>MF: $C_{23}H_{29}O_7$<br>MW: 416.1916 g/mol	Antifungal activities against *F. oxysporum* and *Colletotrichum musae* were observed, with MIC values of 50 and 50 μg/mL, respectively (positive control was triadimefon, with MIC values of 100 and 80 μg/mL, respectively).	*Epicoccum sorghinum* L28	*M. bontioides*	Leizhou Peninsula, China	[108]
**(29) Epicoccether E**<br><br>MF: $C_{32}H_{43}O_7$<br>MW: 538.3014 g/mol	Antifungal activities against *F. oxysporum* and *Colletotrichum musae* were observed, with MIC values of 100 and 50 μg/mL, respectively (positive control was triadimefon, with MIC values of 100 and 80 μg/mL, respectively).	*Epicoccum sorghinum* L28	*M. bontioides*	Leizhou Peninsula, China	[108]
**(30) Epicoccether F**<br><br>MF: $C_{34}H_{50}O_9$	Antifungal activities against *F. oxysporum* and *Colletotrichum musae* were	*Epicoccum sorghinum* L28	*M. bontioides*	Leizhou Peninsula, China	[108]

**Compound Number and Name**	**Associated Biological Activity**[¥]	**Antifungal Producing Endophytic and/or Rhizospheric Species**	**Host Plant/ Source**	**Location**	**Reference**
MW: 602.3349 g/mol	observed, with MIC values of 50 and 100 µg/mL, respectively (positive control was triadimefon, with MIC values of 100 and 80 µg/mL, respectively).				
**(31) Epicoccether G**<br><br>MF: $C_{36}H_{52}O_9$<br>MW: 628.3506 g/mol	Antifungal activities against *F. oxysporum* and *Colletotrichum musae* were observed, with MIC values of 25 and 100 µg/mL, respectively (positive control was triadimefon, with MIC values of 100 and 80 µg/mL, respectively).	*Epicoccum sorghinum* L28	*M. bontioides*	Leizhou Peninsula, China	[108]
**(32) Epicoccether K**<br><br>MF: $C_{18}H_{20}O_7$<br>MW: 348.1130 g/mol	Antifungal activities against *Penicillium italicum* and *F. graminearum* were observed, with MIC values of 100 and 200 µg/mL, respectively (positive control was triadimefon, with MIC values of 50 and 150 µg/mL, respectively).	*Epicoccum sorghinum* L28	*M. bontioides*	Leizhou Peninsula, China	[109]
**(33) Epicoccether L**<br><br>MF: $C_{19}H_{22}O_8$	Antifungal activities against *Penicillium italicum* and *F. graminearum* were observed,	*Epicoccum sorghinum* L28	*M. bontioides*	Leizhou Peninsula, China	[109]

**Compound Number and Name**	**Associated Biological Activity¥**	**Antifungal Producing Endophytic and/or Rhizospheric Species**	**Host Plant/ Source**	**Location**	**Reference**
MW: 378.1390 g/mol	with MIC values of 50 µg/mL for both pathogens (positive control was triadimefon, with MIC values of 50 and 150 µg/mL, respectively).				
**(34) Epicoccether M**<br><br>MF: $C_{20}H_{22}O_8$<br>MW: 390.1391 g/mol	Antifungal activities against *Penicillium italicum* and *F. graminearum* were observed, with MIC values of 50 and 100 µg/mL, respectively (positive control was triadimefon, with MIC values of 50 and 150 µg/mL, respectively).	*Epicoccum sorghinum* L28	*M. bontioides*	Leizhou Peninsula, China	[109]
**(35) Epicoccether N**<br><br>MF: $C_{24}H_{28}O_{10}$<br>MW: 476.1758 g/mol	Antifungal activities against *Penicillium italicum* and *F. graminearum* were observed, with MIC values of 25 and 50 µg/mL, respectively (positive control was triadimefon, with MIC values of 50 and 150 µg/mL, respectively).	*Epicoccum sorghinum* L28	*M. bontioides*	Leizhou Peninsula, China	[109]
**Cyclohexanone and cyclopentenone**					
**(36) Insuetone A**<br><br>MF: $C_{10}H_{16}O_3$<br>MW: 184.11 g/mol	Antifungal activity against *F. oxysporum* and *Colletotrichum gloeosporioides* was observed, with MIC values of 50 and 100 µg/mL, respectively (positive	*Aspergillus insuetus* SYSU6925	Seagrass sp.	Zhuhai, Guangdong Province, China	[111]

Compound Number and Name	Associated Biological Activity¥	Antifungal Producing Endophytic and/or Rhizospheric Species	Host Plant/ Source	Location	Reference
	control was triadimefon, with MIC values of 100 and 50 µg/mL, respectively).				
**(37) Trichodermester A**<br><br>MF: $C_9H_{10}O_4$<br>MW: 182.0497 g/mol	Antifungal activity against *Pestalotiopsis theae* was observed, with an MIC value of 125 mg/disc (positive control hexaconazole was used; MIC value was not reported).	*Trichoderma atroviride* H548	Mangrove sediments	Fujian province, China	[112]
**(38) (±)-(4S*,5S*)-2,4,5-trihydroxy-3-methoxy-4-methoxycarbonyl-5-methyl-2-cyclopenten1-one**<br><br>MF: $C_9H_{12}O_7$<br>MW: 232.0571 g/mol	Antifungal activities against *F. graminearum*, *Colletotrichum musae* were observed, with MIC values of 50 and 200 µg/mL, respectively (positive control was triadimefon, with MIC values of 150 and 100 µg/mL, respectively).	*Alternaria* sp.	Root of a marine semi-mangrove plant *M. bontioides*	Leizhou Peninsula, China	[113]
**Xanthones**					
**(39) 4-chloro-1,5-dihydroxy-3-hydroxymethyl-6methoxycarbonyl-xanthen-9-one**	Antifungal activities against *F. graminearum* and *Colletotrichum musae* were observed, with MIC values of 37.5 and 75 µg/mL,	*Alternaria* sp.	Root of a marine semi-mangrove plant *M. bontioides*	Leizhou Peninsula, China	[113]

Compound Number and Name	Associated Biological Activity¥	Antifungal Producing Endophytic and/or Rhizospheric Species	Host Plant/ Source	Location	Reference
MF $C_{16}H_{10}O_7Cl$<br>MW: 350.0124 g/mol	respectively (positive control was triadimefon, with MIC values of 150 and 100 µg/mL, respectively)				
**(41)**<br>**Aflaxanthone A**<br><br>MF: $C_{30}H_{30}O_{11}$<br>MW: 566.1866 g/mol	Antifungal activities against *Colletotrichum gloeosporioides*, *F. oxysporum*, and *C. albicans* were observed, with MIC values of 1.8, 7.1, 7.1 µg/mL, respectively (positive control was ketoconazole, with MIC values of 0.05, 3.32, 0.05 µg/mL, respectively).	*Aspergillus flavus* QQYZ	*K. candel*	Huizhou, Guangdong province, China	[118]
**(42)**<br>**Aflaxanthone B**<br><br>MF: $C_{30}H_{30}O_{11}$<br>MW: 566.1776 g/mol	Antifungal activities against *F. oxysporum*, *Colletotrichum musae*, and *C. albicans* were observed, with MIC values of 7.1, 7.1, 14.15 µg/mL, respectively (positive control was ketoconazole, with MIC values of 3.32, 0.83, 0.05 µg/mL, respectively).	*Aspergillus flavus* QQYZ	*K. candel*	Huizhou, Guangdong province, China	[118]
**Alkaloids**					
**(43)**<br>**(3aS,8aS)-1-acetyl-1,2,3,3a,8,8a-**	Antifungal activities against *F. oxysporum*, *Penicillium italicum*, and *F. graminearum*	*Botryosphaeria ramosa* L29	Leaf of *M. bontioides*	Leizhou Peninsula, China	[101]

**Compound Number and Name**	**Associated Biological Activity¥**	**Antifungal Producing Endophytic and/or Rhizospheric Species**	**Host Plant/ Source**	**Location**	**Reference**
hexahydropyrrolo[2,3b]indol-3a-ol<br><br>MF: $C_{12}H_{14}N_2O_2$<br>MW: 218.11 g/mol	were observed, with MIC values 6.24, 12.5 and 6.24 µg/mL, respectively (positive control was triadimefon, with MIC values of 100, 50, and 150 µg/mL, respectively).				
**(44) Penicibrocazine B**<br><br>MF: $C_{19}H_{22}N_2O_5S$<br>MW: 390.1249 g/mol	Antifungal activity against *Gaeumannomyces graminis* was observed, with an MIC value of 0.25 µg/mL (positive control was amphotericin B, with an MIC value of 16.0 µg/mL).	*Penicillium brocae* MA-231	*Avicennia marina*	Hainan Island, China	[120]
**(45) Penicibrocazine D**<br><br>MF: $C_{20}H_{26}N_2O_6S_2$<br>MW: 454.1232 g/mol	Antifungal activity against *G. graminis* was observed, with an MIC value of 0.8 µg/mL (positive control was amphotericin B, with an MIC value of 16.0 µg/mL).	*Penicillium brocae* MA-231	*Avicennia marina*	Hainan Island, China	[120]
**(46) Penicibrocazine E**<br>MF: $C_{20}H_{24}N_2O_6S_2$<br>MW: 452.1076 g/mol	Antifungal activity against *G. graminis* was observed, with an MIC value of 0.25 µg/mL (positive control was amphotericin B, with an MIC value of 16.0 µg/mL).	*Penicillium brocae* MA-231	*Avicennia marina*	Hainan Island, China	[120]

Compound Number and Name	Associated Biological Activity¥	Antifungal Producing Endophytic and/or Rhizospheric Species	Host Plant/ Source	Location	Reference
**(49) Brocapyrrozin A**<br><br>MF: $C_{16}H_{19}NO_5$<br>MW: 305.1330 g/mol	Antifungal activity against *F. oxysporum* was observed, with an MIC value of 0.25 µg/mL (positive control was Zeocin, with an MIC value of 0.5 µg/mL).	*Penicillium brocae* MA-231	*Avicennia marina*	Hainan Island, China	[123]
**(50) Brocapyrrozin B**<br><br>MF: $C_{13}H_{15}NO_3$<br>MW: 233.1126 g/mol	Antifungal activity against *F. oxysporum* was observed, with an MIC value of 64.0 µg/mL (positive control was Zeocin, with an MIC value of 0.5 µg/mL).	*Penicillium brocae* MA-231	*Avicennia marina*	Hainan Island, China	[123]
	Antifungal activity against *R. solani* was observed, with an MIC values of 100 µg/mL (positive control was carbendazim, with an MIC value of 6.25 µg/mL).	*Penicillium chrysogenum* V11	A leaf vein of *M. bontioides*	Leizhou Peninsula, China	[124]
**(52) Penochalasin J**<br><br>MF: $C_{32}H_{38}N_2O_3$<br>MW: 498.2963 g/mol	Antifungal activity against *Colletotrichum musae*, *Colletotrichum gloeosporioides* (Penz) Sacc., *Penicillium italicum*, and *R. solani* was observed, with MIC values of 50, 50, 12.5, and 25.08 µg/mL, respectively (positive control was carbendazim, with MIC	*Penicillium chrysogenum* V11	Vein of *M. bontioides* A. Gray	Leizhou Peninsula, China	[124]

**Compound Number and Name**	**Associated Biological Activity**¥	**Antifungal Producing Endophytic and/or Rhizospheric Species**	**Host Plant/ Source**	**Location**	**Reference**
	values of 6.25, 3.12, 6.25, and 12.5 µg/mL, respectively).				
**(53) Penochalasin K**<br><br>MF: $C_{32}H_{34}N_2O_4$<br>MW: 510.25 g/mol	Antifungal activity against *F. oxysporum* and *Colletotrichum gloeosporioides* was observed, with MICs values of 100 and 50 µg/mL, respectively (positive control was triadimefon, with MIC values of 100 and 50 µg/mL, respectively).	*Penicillium chrysogenum* V11	A leaf vein of *M. bontioides*	Leizhou Peninsula, China	[125]
**Terpenes**					
**(57) Ethyl hydroheptelidate**<br><br>MF: $C_{17}H_{26}O_6$<br>MW: 326.18 g/mol	Antifungal activity against F, oxysporum and *Collerotichum gloeosporioides* was observed, with MIC values of 100 and 50 µg/mL, respectively (positive control was triadimefon, with MIC values of a 100 and 50 µg/mL, respectively).	*Trichoderma harzianum*	*M. bontioides*	Leizhou Peninsula, China	[128]
**(58) Asperalacid A**<br><br>MF: $C_{15}H_{22}O_4$<br>MW: 266.1443 g/mol	Antifungal activity against *F. graminearum* was observed, with an MIC value of 200 µg/mL (positive control was triadimefon, with an MIC value of a 150 µg/mL).	*Aspergillus alabamensis* SYSU-6778	Necrotic leaves and normal roots of *Enhalus acoroides*	Hainan Island, China	[94]

Compound Number and Name	Associated Biological Activity¥	Antifungal Producing Endophytic and/or Rhizospheric Species	Host Plant/ Source	Location	Reference
**(59) Asperalacid B**<br><br>MF: $C_{15}H_{21}O_4$<br>MW: 266.1443 g/mol	Antifungal activity against *F. oxysporum* and *F. graminearum* was observed, with MIC values of 100 and 200 µg/mL, respectively (positive control was triadimefon, with MIC values of 100 and 150 µg/mL, respectively).	*Aspergillus alabamensis* SYSU-6778	Necrotic leaves and normal roots of *Enhalus acoroides*	Hainan Island, China	[94]
**(60) Asperalacid C**<br><br>MF: $C_{15}H_{22}O_5$<br>MW: 282.1393 g/mol	Antifungal activity against *F. oxysporum* and *F. graminearum* was observed, with MIC values of 100 and 25 µg/mL, respectively (positive control was triadimefon, with MIC values of 100 and 150 µg/mL respectively).	*Aspergillus alabamensis* SYSU-6778	Necrotic leaves and normal roots of *Enhalus acoroides*	Hainan Island, China	[94]
**(61) Asperalacid D**<br><br>MF: $C_{15}H_{20}O_4$<br>MW: 264.1292 g/mol	Antifungal activity against *F. graminearum* was observed, with an MIC value of 200 µg/mL (positive control was triadimefon, with an MIC value of 150 µg/mL).	*Aspergillus alabamensis* SYSU-6778	Necrotic leaves and normal roots of *Enhalus acoroides*	Hainan Island, China	[94]
**(62) 4-hydroxy-5(6)-dihydroterrecyclic acid A**	Antifungal activity against *F. oxysporum*, *F. graminearum*, and *Penicillium italicum* were observed, with MIC values of 100, 50, and 200 µg/mL,	*Aspergillus alabamensis* SYSU-6778	Necrotic leaves and normal roots of *Enhalus acoroides*	Hainan Island, China	[94]

**Compound Number and Name**	**Associated Biological Activity**[¥]	**Antifungal Producing Endophytic and/or Rhizospheric Species**	**Host Plant/ Source**	**Location**	**Reference**
MF: $C_{15}H_{24}O_3$<br>MW: 252.1653 g/mol	respectively (positive control was triadimefon, with MIC values of 100, 150, and 50 μg/mL, respectively).				
**Other compounds**					
**(63) Peniprenylphenol A**<br>MF: $C_{13}H_{20}O_5$<br>MW: 256.1386 g/mol	Antifungal activity against *C. albicans* was observed, with an MIC value of 13 μg/mL (positive control was amphotericin B, with an MIC value of 3.0 μg/mL).	*Penicillium chrysogenum* ZZ1151	Mangrove vegetated tidal flat/sediment	Pangkep District South Sulawesi province, Indonesia	[129]
**(64) Cordyanhydride A ethyl ester**<br>MF:C22H26O8<br>MW: 418.1701 g/mol	Antifungal activity against *Botrytis cinerea*, *F. graminearum*, *F. oxysporum*, and *R. solani* was observed, with MIC values of 12.5, 6.25, 6.25, and 6.25 μg/mL, respectively (positive control was cycloheximide, with MIC values of 12.5, 12.5, 25, and 25 μg/mL, respectively).	*Talaromyces* sp. SCSIO 41050	Mangrove sediment sample	Gaoqiao mangrove wetland, Zhanjiang, coastline of the northern part of Beibu Gulf, China	[130]
**(67) Tandyukisin J**<br>MF: $C_{25}H_{38}O_7$	Antifungal activities against *C. albicans* and *Penicillium italicum* were observed, with	*Trichoderma lentiforme* ML-P8-2	Fresh leaf of *B. gymnorrhiza*	Dongzhaigan g	[96]

Compound Number and Name	Associated Biological Activity¥	Antifungal Producing Endophytic and/or Rhizospheric Species	Host Plant/ Source	Location	Reference
MW: 450.2609	MIC values of 11.26 and 2.81 µg/mL, respectively (positive control was ketoconazole, with MIC values of 0.07 and 0.83 µg/mL, respectively).			National Nature Reserve in Hainan Province, China	
**(69) Isobisvertinol A**<br><br>MF: $C_{28}H_{35}O_8$<br>MW: 498.2312 g/mol	Antifungal activity against *Pestalotiopsis theae* was observed, with an MIC value of 9.13 µg/mL (positive control was hexaconazole, with an MIC value of 24.25 µg/mL).	*Hypocrea jecorina H8*	Mangrove sediment	Fujian province, China	[131]
**(71) (5S, 8R)-simplicilopyrone**<br><br>MF: $C_9H_{14}O_4$<br>MW: 186.0966 g/mol	Antifungal activity against *F. oxysporum*, *Colletotrichum musae* Arx, and *F. graminearum* was observed, with MIC values of 50, 50, and 200 µg/mL, respectively (positive control was triadimefon, with MIC values of 100, 80, and 150 µg/mL, respectively).	*Botryosphaeria ramosa* L29	Leaf of *M. bontioides*	Leizhou Peninsula, China	[97]

**Compound Number and Name**	**Associated Biological Activity**[¥]	**Antifungal Producing Endophytic and/or Rhizospheric Species**	**Host Plant/ Source**	**Location**	**Reference**
**(72) Botroxepinone**<br><br>MF: $C_9H_{12}O_4$<br>MW: 184.0811 g/mol	Antifungal activity against *F. oxysporum*, *Colletotrichum musae*, and *F. graminearum* was observed, with MIC values of 200, 25, and 200 μg/mL, respectively (positive control was triadimefon, with MIC values of 100, 80, and 150 μg/mL, respectively).	*Botryosphaeria ramosa* L29	Leaf of *M. bontioides*	Leizhou Peninsula, China	[97]
**(73) Nafuredin C**<br><br>MF: $C_{22}H_{34}O_4$<br>MW: 362.245 g/mol	Antifungal activity against *Magnaporthe oryzae* was observed, with an MIC value of 3.13 μg/mL (positive control was carbendazim, with an MIC value of 0.625 μg/mL).	*Trichoderma harzianum* D13.	Root of mangrove plant *Excoecaria agallocha* Linn.	Hainan province, China	[133]
**(75) 3-hydroxy-5-methoxy-2,4,6-trimethylbenzoic acid**<br><br>MF: $C_{11}H_{14}O_4$<br>MW: 210.0892 g/mol	Antifungal activity against *C. albicans* was observed, with an MIC value of 2.62 μg/mL (positive control was ketoconazole, with an MIC value of 0.1 μg/mL).	*Phoma* sp. SYSU-SK-7	*K. candel* healthy branch	Guangxi Province, China	[135]

* Molecular formula
# Molecular weight

¥When the novel compound's antifungal activity is equal to or exceeds the antifungal activity of the tested positive control against the respective pathogenic fungi (indicator species), the respective indicator species is coloured in red. When the novel compound's antifungal activity is less than the antifungal activity of the tested positive control against the respective pathogenic fungi (indicator species), the respective indicator species is coloured in green.

Table S2. Antifungal compounds discovered from endophytic and rhizospheric bacteria including Actinomycetes associated with coastal vegetation.

**Compound Number and Name**	**Associated Biological Activity¥**	**Antifungal Producing Endophytic and/or Rhizospheric Species**	**Source Host**	**Location**	**Reference**
**Macrolides**					
**(76) Antifungalmycin B**<br>MF: C33H56O14<br>MW: 676.3578 g/mol	Antifungal activity against *Talaromyces marneffei* was observed, with an MIC value of 16 µg/mL (positive controls were fluconazole and amphotericin B, with MIC values of 16 and 0.5 µg/mL, respectively).	*Streptomyces hiroshimensis* GXIMD 06359	Mangrove	West coast, Hainan, China	[136]
**(77) Antifungalmycin E**<br>MF: C35H60O13<br>MW: 688.3975 g/mol	Antifungal activity against *Talaromyces marneffei* was observed, with an MIC value of 32 µg/mL (positive controls were fluconazole and	*Streptomyces hiroshimensis* GXIMD 06359	Mangrove	West coast, Hainan, China	[136]

**Compound Number and Name**	**Associated Biological Activity**¥	**Antifungal Producing Endophytic and/or Rhizospheric Species**	**Source Host**	**Location**	**Reference**
	amphotericin B, with MIC values of 16 and 0.5 μg/mL, respectively).				
**(78)**<br>**Fungichromin B (6′-methyl-fungichromin)**<br><br>MF: $C_{36}H_{60}O_{12}$<br>MW: 684 g/mol	Antifungal activity against *Saccharomyces cerevisiae, F. oxysporum* and *Aspergillus niger* showed 14 mm, 12 mm, and 15 mm inhibition zones for 0.10 μg/<br>6 mm disc (no positive control recorded).	*Streptomyces albogriseolus* HA10002	Mangrov e sediment	Hainan, China	[137]
**(80)**<br>**25-malonyl demalonylazalomycin F5a monoester**<br><br>MF: $C_{57}H_{97}N_3O_{17}$<br>MW: 1095.69 g/mol	Antifungal activity against *Candida albicans* ATTC 10231 was observed, with an MIC value of 3.13 μg/mL (positive control was amphotericin B, with an MIC value of 2.0 μg/mL).	*Streptomyces* sp. 211726	Mangrov e *Heritiera globosa* rhizosphe re soil	Wenchang, China	[140]
**(81)**	Antifungal activity against *C. albicans* ATTC 10231 was observed,	*Streptomyces* sp. 211726	Mangrov e *Heritiera globosa*	Wenchang, China	[140]

**Compound Number and Name**	**Associated Biological Activity¥**	**Antifungal Producing Endophytic and/or Rhizospheric Species**	**Source Host**	**Location**	**Reference**
**23-valine demalonylazalomycin F5a ester**<br><br>MF: $C_{59}H_{104}N_4O_{15}$<br>MW: 1108.76 g/mol	with an MIC value of 6.25 µg/mL (positive control was amphotericin B, with an MIC value of 2.0 µg/mL).		rhizosphere soil		
**(82)**<br>**23-(6-methyl)heptanoic acid demalonylazalomycins F3a ester**<br><br>MF: $C_{60}H_{105}N_3O_{15}$<br>MW: 1107.76 g/mol	Antifungal activity against *C. albicans* ATTC 10231 was observed, with an MIC value of 3.13 µg/mL (positive control was amphotericin B, with an MIC value of 2.0 µg/mL).	*Streptomyces* sp. 211726	Mangrove *Heritiera globosa* rhizosphere soil	Wenchang, China	[140]
**(83)**<br>**Azalomycin F analogue 4 (F4a ester)**<br><br>MF: $C_{61}H_{107}N_3O_{15}$<br>MW: 1121.78 g/mol	Antifungal activity against *C. albicans* ATCC 10231 was observed, with an MIC value of 1.56 µg/mL (positive control was amphotericin B, with an MIC value of 2.0 µg/mL).	*Streptomyces* sp. 211726	Mangrove *Heritiera globosa* rhizosphere soil	Wenchang, China	[140]
**(84)**<br>**Azalomycin F analogue 5 (F5a ester)**	Antifungal activity against *C. albicans* ATCC 10231 was observed, with an MIC value of	*Streptomyces* sp. 211726	Mangrove *Heritiera globosa*	Wenchang, China	[140]

**Compound Number and Name**	**Associated Biological Activity[¥]**	**Antifungal Producing Endophytic and/or Rhizospheric Species**	**Source Host**	**Location**	**Reference**
MF: $C_{62}H_{109}N_3O_{15}$<br>MW: 1135.80 g/mol	1.56 µg/mL (positive control was amphotericin B, with an MIC value of 2.0 µg/mL).		rhizosphere soil		
**(85)**<br>**23-(9-methyl)decanoic acid demalonylazalomycin F4a ester**<br><br>MF: $C_{64}H_{113}N_3O_{15}$<br>MW: 1163.83 g/mol	Antifungal activity against *C. albicans* ATTC 10231 was observed, with an MIC value of 3.13 µg/mL (positive control was amphotericin B, with an MIC value of 2.0 µg/mL).	*Streptomyces* sp. 211726	Mangrove *Heritiera globosa* rhizosphere soil	Wenchang, China	[140]
**(86)**<br>**23-(10-methyl)undecanoic acid demalony lazalomycin F4a ester**<br><br>MF: $C_{65}H_{115}N_3O_{15}$<br>MW: 1177.84 g/mol	Antifungal activity against *C. albicans* ATTC 10231 was observed, with an MIC value of 3.13 µg/mL (positive control was amphotericin B, with an MIC value of 2.0 µg/mL).	*Streptomyces* sp. 211726	Mangrove *Heritiera globosa* rhizosphere soil	Wenchang, China	[140]
**Other compounds**					

**Compound Number and Name**	**Associated Biological Activity**[¥]	**Antifungal Producing Endophytic and/or Rhizospheric Species**	**Source Host**	**Location**	**Reference**
**(87)**<br>**7,30-di-(g,g-dimethylallyloxy)-5-hydroxy-40**<br>**-methoxyflavone**<br><br>MF: $C_{26}H_{28}O_6$<br>MW: 436.1886 g/mol	Antifungal activities (inhibitory zone diameters) against *Colletotrichum musae, Gibberella zeae* (Schweinitz) Petch, and *Penicillium citrinum* Thom<br>were observed, with 12.70, 13.00, and 12.17 mm zones, respectively, at a concentration of 0.25 mM of the compound.	*Streptomyces* sp. strain MA-12	Semi-mangrove plant *Myoporum bontioides* A. Gray	Guangdong province, China	[142]

* Molecular formula

[#] Molecular weight

[¥]When the novel compound's antifungal activity is equal to or exceeds the antifungal activity of the tested positive control against the respective pathogenic fungi (indicator species), the respective indicator species is coloured in red. When the novel compound's antifungal activity is less than the antifungal activity of the tested positive control against the respective pathogenic fungi (indicator species), the respective indicator species is coloured in green.
